# Novel Insight into the Prevention and Therapeutic Treatment of *Paulownia* Witches’ Broom: A Study on the Effect of Salicylic Acid on Disease Control and the Changes in the *Paulownia* Transcriptome and Proteome

**DOI:** 10.3390/ijms251910553

**Published:** 2024-09-30

**Authors:** Yujie Fan, Peipei Zhu, Hui Zhao, Haibo Yang, Wenhu Wang, Guoqiang Fan

**Affiliations:** 1 College of Forestry, Henan Agricultural University, Zhengzhou 450002, China; fanyujie@henau.edu.cn (Y.F.); 13730357685@163.com (P.Z.); zhao_hui_zh@163.com (H.Z.); haiboyang@henau.edu.cn (H.Y.); wwh15693348309@126.com (W.W.); 2 Institute of Paulownia, Henan Agricultural University, Zhengzhou 450002, China

**Keywords:** *Paulownia fortunei*, *Paulownia tomentosa*, *Paulownia* witches’ broom, salicylic acid, transcriptomic, proteomic

## Abstract

*Paulownia* species not only have significant economic benefits but also show great potential in ecological conservation. However, they are highly susceptible to phytoplasma infections, causing *Paulownia* witches’ broom (PaWB), which severely restricts the development of the *Paulownia* industry. Salicylic acid (SA) plays a crucial role in plant disease resistance. However, there have been no reports on the effect of SA on PaWB. Due to the properties of SA, it may have potential in controlling PaWB. Based on the above speculation, the prevention and therapeutic effect of SA on PaWB and its effect on the PaWB-infected *Paulownia* transcriptome and proteome were studied in this work. The results indicated that 0.1 mmol/L was the optimal SA concentration for inhibiting the germination of *Paulownia* axillary buds. In terms of resistance physiological indicators, SA treatment significantly affected both *Paulownia tomentosa* infected (PTI) seedlings and *Paulownia fortunei* infected (PFI) seedlings, where the activities of peroxidase (POD) and superoxide dismutase (SOD) were enhanced. Malondialdehyde (MDA), O_2_^−^, and H_2_O_2_, however, were significantly reduced. Specifically, after SA treatment, SOD activity increased by 28% in PFI and 25% in PTI, and POD activity significantly increased by 61% in PFI and 58% in PTI. Moreover, the MDA content decreased by 30% in PFI and 23% in PTI, the H_2_O_2_ content decreased by 26% in PFI and 19% in PTI, and the O_2_^−^ content decreased by 21% in PFI and 19% in PTI. Transcriptomic analysis showed that there were significant upregulations of MYB, NAC, and bHLH and other transcription factors after SA treatment. Moreover, genes involved in PaWB-related defense responses such as RAX2 also showed significant differences. Furthermore, proteomic analysis indicated that after SA treatment, proteins involved in signal transduction, protein synthesis modification, and disease defense were differentially expressed. This work provides a research foundation for the prevention and treatment of PaWB and offers references for exploring anti-PaWB methods.

## 1. Introduction

Paulownia species are renowned for their rapid growth and wide adaptability [1,2]. Paulownia species have a wide range of uses, but they are prone to infection by phytoplasma during their growth, leading to *Paulownia* witches’ broom (PaWB) [3]. Phytoplasma is a unique class of bacterial pathogens that specifically parasitize the phloem of host plants, causing a range of typically morphological abnormalities such as the proliferation of axillary buds, stunted growth, and leaf yellowing, with severe infections potentially leading to plant death [4,5]. Globally, there are over a thousand plant diseases caused by phytoplasma infections, such as strawberry yellowing disease [6], grapevine yellows [7], and onion yellows [8], which cause significant economic losses to agriculture [9]. PaWB is highly contagious and harmful, with high mortality rates and difficult recovery, causing severe losses to the Paulownia-related industries. Therefore, the prevention and treatment of PaWB has become one of the key contents of Paulownia research.

The endogenous hormones of Paulownia species will also be disturbed after infection by phytoplasma [10]. Salicylic acid (SA), a phenolic small molecule compound that is widely present in plants, influences seed germination, stomatal movement, transpiration rate, and the biosynthesis of secondary metabolites, demonstrating its multifaceted role in stress responses, plant growth, and development [11,12]. Research studies have indicated that exogenous SA treatment or increased endogenous SA levels can significantly enhance plant resistance to various biotic stresses [13]. Further studies have shown that SA mainly activates the expression of disease-related genes through key transcription factors and initiates broad-spectrum resistance in plants [14]. As an important signaling molecule in plant disease resistance, SA activates defense mechanisms including the hypersensitive reaction (HR) and systemic acquired resistance (SAR), inducing the expression of various pathogenesis-related proteins, thereby enhancing plant resistance to pathogens. The application of SA has been shown to improve apple resistance to leaf spot disease [15]. When plants are induced by exogenous SA, the activity of related defense enzymes significantly increases, enhancing plant disease resistance. Additionally, studies have found that exogenous SA can suppress the occurrence of clubroot disease in cabbage [16], indicating the broad-spectrum role of SA in plant disease resistance. These findings indicate that SA may have potential in the prevention and treatment of PaWB.

In recent years, the continuous development and innovation of omics technologies have brought unprecedented opportunities for the prevention and therapeutic of plant diseases [17,18]. These technologies not only support the breeding of disease-resistant varieties but also greatly promote the rapid detection and effective control of pathogens [19,20]. Omics techniques such as transcriptomics [21,22], metabolomics [23], and proteomics [24] have been applied in the study of PaWB. Researchers have identified differentially expressed genes involved in plant–pathogen interactions through transcriptome sequencing of Paulownia affected by PaWB [25]. These research efforts on PaWB provide a research foundation for its prevention and control. While the role of SA in plant biotic and abiotic stress responses has been extensively studied and validated in various plants, its application and research in the prevention and treatment of PaWB have not yet been reported. Based on the above-mentioned facts and speculation, this work studied the effects of SA on the prevention and treatment of PaWB and preliminarily explored its inhibitory effects on PaWB through combined transcriptomic and proteomic analysis.

## 2. Results

### 2.1. Effects of SA Treatment on the Growth Morphology of Paulownia Seedlings

*Paulownia fortunei* infected (PFI) and *Paulownia tomentosa* infected (PTI) seedlings were treated with different concentrations of SA and the optimal concentration was determined by comparing the morphological changes after treatment and without treatment for PF (*Paulownia fortunei*) and PT (*Paulownia tomentosa*). The results showed that 0.10 mmol/L SA could not only inhibit the axillary bud growth of Paulownia but also avoid affecting the normal growth of Paulownia.

Under 0.10 mmol/L, both PTI and PFI seedlings showed a good growth situation, and the inhibitory effect on plant growth was inconspicuous. This phenomenon indicated that 0.10 mmol/L SA was the optimal concentration for controlling PaWB in PFI and PTI seedlings (Supplementary Figures S1 and S2). The subsequent omics experiments adopted 0.10 mmol/L as the treatment concentration of SA.

### 2.2. Effects of SA Treatment on Physiological Indicators of Paulownia Seedlings

**Figure 1:**
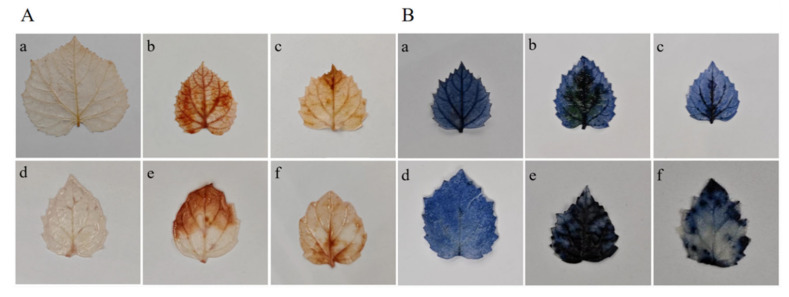
(**A**) H_2_O_2_ content under DAB staining. (**a**) PF. (**b**) PFI. (**c**) PFI + 0.1 mM SA treatment. (**d**) PT. (**e**) PTI. (**f**) PTI + 0.1 mM SA treatment. (**B**) O_2_^−^ content under NBT staining. (**a**) PF. (**b**) PFI. (**c**) PFI + 0.1 mM SA treatment. (**d**) PT. (**e**) PTI. (**f**) PTI + 0.1 mM SA treatment.

**Figure 2:**
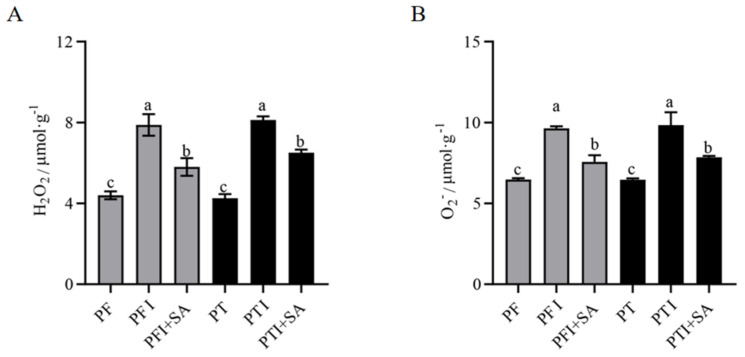
Quantitative determination of (**A**) H_2_O_2_ content and (**B**) O_2_^−^ content. The different letters (a, b, c) on the bars in the figure indicate significant differences at the 5% level (*p* ˂ 0.05).

Leaves from healthy seedlings of both PF and PT showed no brown or blue substances (Figure 1). However, leaves from phytoplasma-infected Paulownia exhibited extensive brown and blue areas, indicating that large amounts of reactive oxygen species (ROS) were produced. The amounts of H_2_O_2_ and O_2_^−^ in leaves were determined using DAB and NBT staining (Figure 2), and both were significantly higher in PFI and PTI compared to PF and PT. After SA treatment, the contents of H_2_O_2_ and O_2_^−^ in PFI and PTI were significantly decreased and were lower than in those not treated with SA (Figure 2).

**Figure 3:**
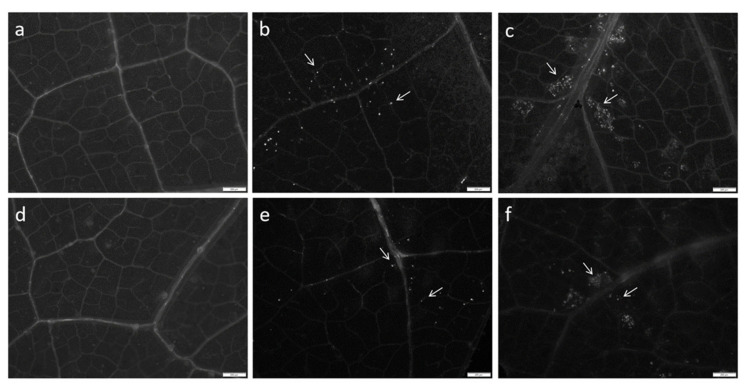
Effect of SA treatment on callose precipitation. (**a**) PF. (**b**) PFI. (**c**) PFI + 0.1 mM SA treatment. (**d**) PT. (**e**) PTI. (**f**) PTI + 0.1 mM SA treatment. Bar = 200 μm. The fluorescence indicated by the white arrow is the callose precipitation.

The SA-based defense response plays an important role in callose deposition in plants [26]. Through tissue staining and fluorescence microscopy, callose deposition in the leaves of different seedlings was observed (Figure 3). In PFI and PTI, callose deposition was noticeable. After SA treatment, the amount of callose deposition in diseased Paulownia seedlings was significantly higher than that in untreated groups (*p* ≤ 0.05). In contrast, very little callose deposition occurred in healthy seedlings. Additionally, the deposition of callose was mainly concentrated near the leaf veins. This indicated that Paulownia initiated a defensive response through callose deposition upon phytoplasma infection, and SA treatment enhanced resistance by increasing callose accumulation.

**Figure 4:**
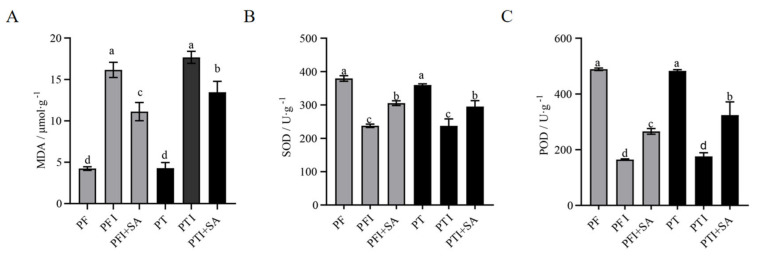
Effects of SA treatment on the content of (**A**) malondialdehyde (MDA), (**B**) superoxide dismutase (SOD), and (**C**) peroxidase (POD). The different letters (a, b, c, d) on the bars in the figure indicate significant differences at the 5% level (*p* ˂ 0.05).

In PFI and in those treated with SA (PFI + SA), the malondialdehyde (MDA) content was significantly higher than in PF. After SA treatment, the MDA content in PFI+SA was higher than in PF but lower than in untreated diseased seedlings (Figure 4A), indicating that SA treatment could reduce lipid peroxidation by lowering the MDA content. The superoxide dismutase (SOD) activity in PFI + SA and PTI + SA was higher than in untreated PFI and PTI, but still lower than in PF (Figure 4B). The trend in peroxidase (POD) content was similar to that of SOD, with increased activity in PFI+SA and PTI + SA compared to untreated PFI and PTI, but still lower than in PF (Figure 4C).

### 2.3. Effects of SA on Phytoplasma

**Figure 5:**
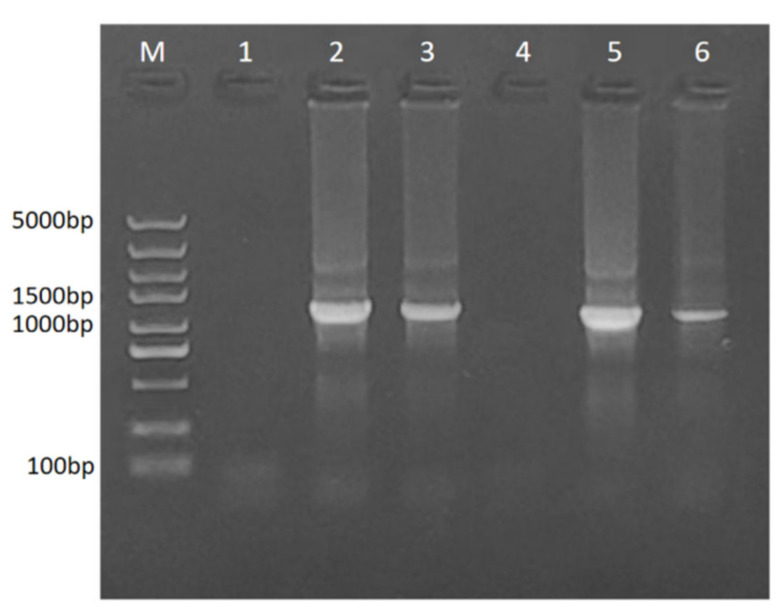
Effect of SA treatment on phytoplasma content. M: DL5000 Marker; 1: PF; 2: PFI; 3: PFI+0.1 mmol/L SA treatment; 4: PT; 5: PTI; 6: PTI+0.1 mmol/L SA.

In this study, the presence of phytoplasma was detected in the apical buds of PF, PFI, PFI + SA, PT, PTI, and PTI + SA to determine the effect of SA on PaWB. The results indicated that PF and PT showed no target bands, while PFI, PFI+SA, PTI, and PTI + SA all showed target bands at 1200 bp. The bands were less intense in PFI+SA and PTI + SA than those in PFI and PTI (Figure 5). This indicated that phytoplasma was present in PFI, PFI+SA, PTI, and PTI + SA, but not in PF and PT. After SA treatment, the infection level was reduced, suggesting that SA had a certain suppressive effect on phytoplasma infection.

### 2.4. Transcriptomic Analysis under SA Treatment

**Figure 6:**
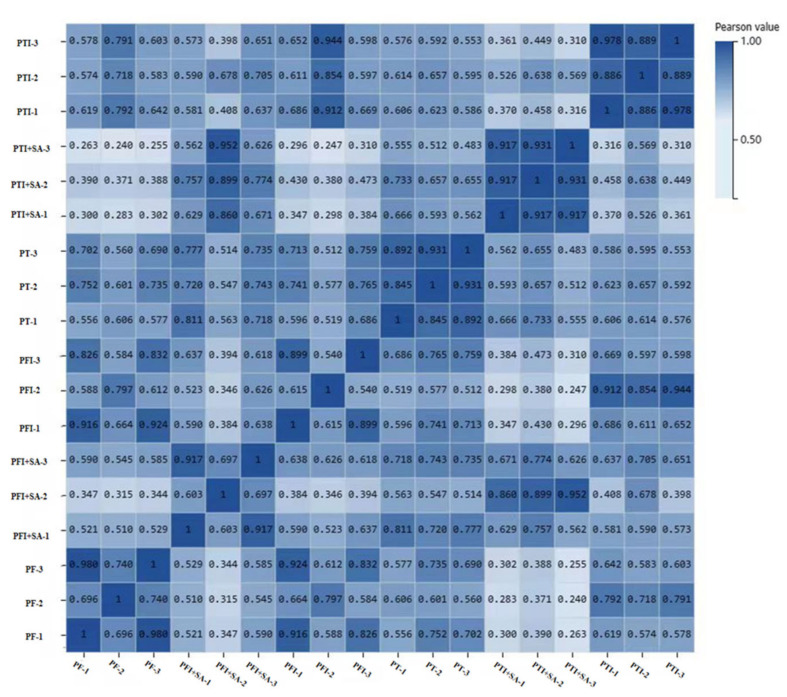
Sample correlation heatmap. PTI: *Paulownia tomentosa* infected; PT: *Paulownia tomentosa*; PFI: *Paulownia fortunei* infected; PF: *Paulownia fortunei*.

Transcriptome sequencing analysis was performed on 18 samples (including three biological replicates), yielding clean reads data (Table 1). A total of 2425 new genes were predicted and 2518 new transcripts were detected. Reproducibility assessment of sequencing data from SA-treated diseased seedlings and controls, as shown in the correlation graph (Figure 6), indicated a high similarity within groups and significant differences between groups.

**Figure 7:**
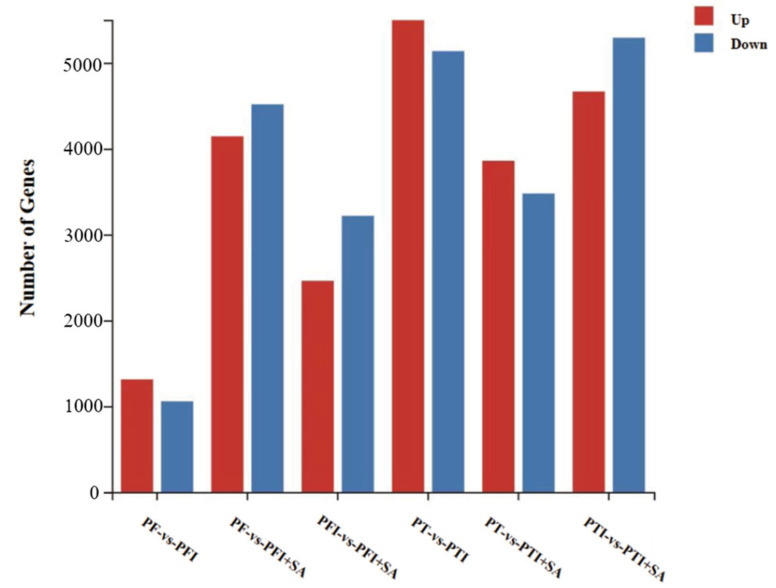
Statistical plot of the number of DEGs (differentially expressed genes). PTI: *Paulownia tomentosa* infected; PT: *Paulownia tomentosa*; PFI: *Paulownia fortunei* infected; PF: *Paulownia fortunei*.

**Figure 8:**
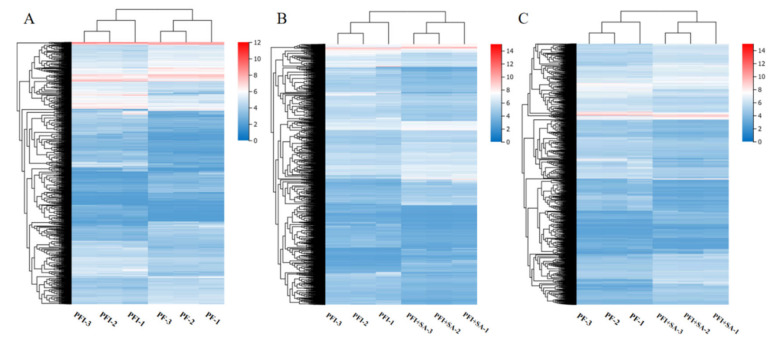
Clustering analysis of DEGs. (**A**) PF vs. PFI. (**B**) PFI vs. PFI+SA treatment. (**C**) PF vs. PFI+SA treatment.

The changes in gene expression were analyzed, and significant differentially expressed genes (DEGs) were found (Figure 7). PF vs. PFI had 2369 DEGs, with 1312 upregulated and 1057 downregulated; PFI vs. PFI+SA had 5677 DEGs, with 2460 upregulated and 3217 downregulated; PF vs. PFI+SA had 8659 DEGs, with 4143 upregulated and 4516 downregulated. GO functional annotation and classification found that 2036, 4824, and 7370 DEGs in PF vs. PFI, PFI vs. PFI+SA, and PF vs. PFI+SA, respectively, had functional annotation information. Cluster analysis of DEGs in each comparison group based on FPKM values is shown in Figure 8. GO classification revealed that all DEGs were annotated to 37, 38, and 40 GO subcategories, respectively (Supplementary Figure S3). The enrichment analysis of DEGs in three comparison groups showed significant enrichment in activities involving paired donor oxidoreductases and iron ion binding in PF vs. PFI; significant enrichment in the cell cycle and chromosomes in PFI vs. PFI+SA; and significant enrichment in the cell cycle in PF vs. PFI+SA (Supplementary Figure S4).

According to KEGG pathway enrichment analysis (Qvalue ≤ 0.05), the DEGs in PF vs. PFI were significantly enriched in plant hormone signal transduction and plant–pathogen interactions; the DEGs in PFI vs. PFI+SA were significantly enriched in circadian rhythm and plant–pathogen interactions; and the DEGs in PF vs. PFI+SA were significantly enriched in plant circadian rhythm and plant–pathogen interactions. The DEGs in SA-treated samples were significantly enriched in flavonoid and anthocyanin biosynthesis (Supplementary Figures S5 and S6). Antioxidant-related genes identified from DEGs were upregulated after SA treatment, which was consistent with the earlier physiological indicator detection result. DEGs comparative analysis identified five key DEGs related to the occurrence of PaWB. After annotation, it was found that these five genes included one cytochrome P450 family (Pfo02g008160) gene, two embryoid protein subfamily members 18 (precursor) genes (Pfo06g007450, Pfo02g008160), one LTP family (Pfoxxg013220), and one thymosin-like protein (precursor) gene (Pfo18g008090).

**Figure 9:**
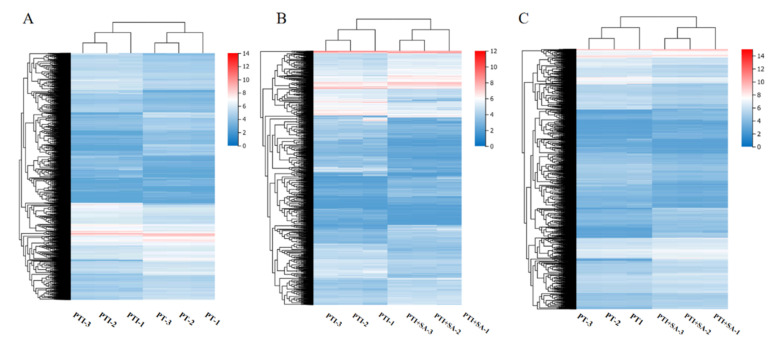
Clustering analysis of DEGs. (**A**) PT vs. PTI. (**B**) PTI vs. PTI + SA treatment. (**C**) PT vs. PTI + SA treatment.

In the PT vs. PTI group, 10,632 DEGs were detected, with 5496 upregulated and 5136 downregulated; in the PTI vs. PTI + SA group, 9957 DEGs were detected, with 4665 upregulated and 5292 downregulated; in the PT vs. PTI + SA group, 7336 DEGs were detected, with 3859 upregulated and 3477 downregulated. These genes were annotated and classified for GO functions in PT vs. PTI, PTI vs. PTI + SA, and PT vs. PTI + SA groups, respectively, with 9038, 8476, and 6307 DEGs having functional annotation information. Cluster analysis of the expression levels of these DEGs is shown in Figure 9. GO classification revealed that DEGs were annotated to 41, 40, and 40 GO subcategories (Supplementary Figure S7).

GO enrichment analysis found that in the PT vs. PTI group, the DEGs were significantly enriched in catalytic activity, followed by cytoplasm, and least in chlorophyll metabolism; in the PTI vs. PTI + SA group, significant enrichment was also found in catalytic activity, followed by oxidation–reduction processes; in the PT vs. PTI + SA group, significant enrichment was found in ATP binding (Supplementary Figure S8). KEGG pathway enrichment analysis revealed that in PT vs. PTI, the DEGs were most enriched in cofactor biosynthesis, followed by plant circadian rhythm, and least in glucosinolate biosynthesis (Supplementary Figures S9 and S10). In PTI vs. PTI + SA, the most enrichment was found in plant hormone signal transduction, followed by the MAPK signaling pathway in plants, with the least enrichment in various alkaloid biosyntheses, only seven instances. In PT vs. PTI + SA, significant enrichment was found in zeatin biosynthesis, the biosynthesis of plant secondary metabolites, and the metabolism of alanine, aspartate, and glutamate. The enrichment results also showed significant enrichment in steroid biosynthesis in the DEGs after SA treatment. Similar to the analysis of PF, no reactive oxygen species metabolism pathways appeared in the top 20 KEGG pathways, and antioxidant-related genes were also selected from the DEGs, all of which were upregulated after SA treatment. Through screening, four key DEGs were identified, namely thioredoxin like 3-1, chloroplast (precursor) (Pfoxxg017100), WCRKC1, and two MYB domain genes RAX2 (Pfoxxg018960 and Pfoxxg018960).

**Figure 10:**
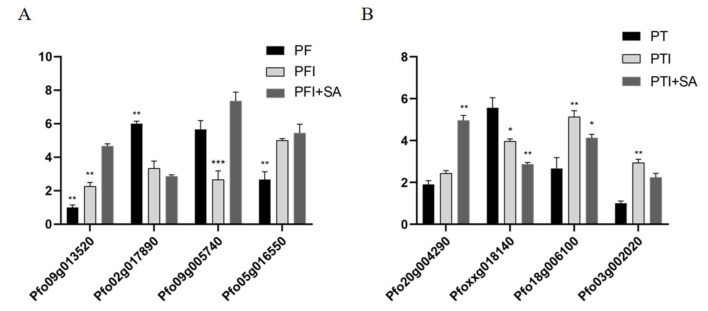
Validation by qRT-PCR. (**A**) Validation of the transcriptome sequencing of PF, PFI, and PFI + SA. (**B**) Validation of the transcriptome sequencing of PT, PTI, and PTI + SA. *, ** and *** stand for *p* < 0.05, *p* < 0.01 and *p* < 0.001, respectively.

In total, four genes were randomly selected from every sample (PF, PFI, PFI+SA, PT, PTI, and PTI + SA) to verify the results of the transcriptome sequencing. As shown in Figure 10, in PFI+SA, the expression of three genes was upregulated and one gene was downregulated; in PTI + SA, one gene was upregulated and three genes were downregulated, which was consistent with the results of the transcriptome sequencing.

### 2.5. Proteomic Analysis under SA Treatment

Total protein was quality checked, and the protein quality of each sample met the requirements for mass spectrometry analysis. Quantitative protein analysis was performed on 18 samples, identifying 53,944 peptides corresponding to 9262 proteins. Based on the differential fold of the proteins (taking log2 values), 7725 proteins were identified in PFI vs. PFI+SA, with 199 proteins upregulated in PFI and 1122 proteins upregulated in PFI+SA. In PTI vs. PTI + SA, a total of 7627 proteins were identified, with 544 proteins upregulated in PTI and 1035 proteins upregulated in PTI + SA (Table 2).

**Figure 11:**
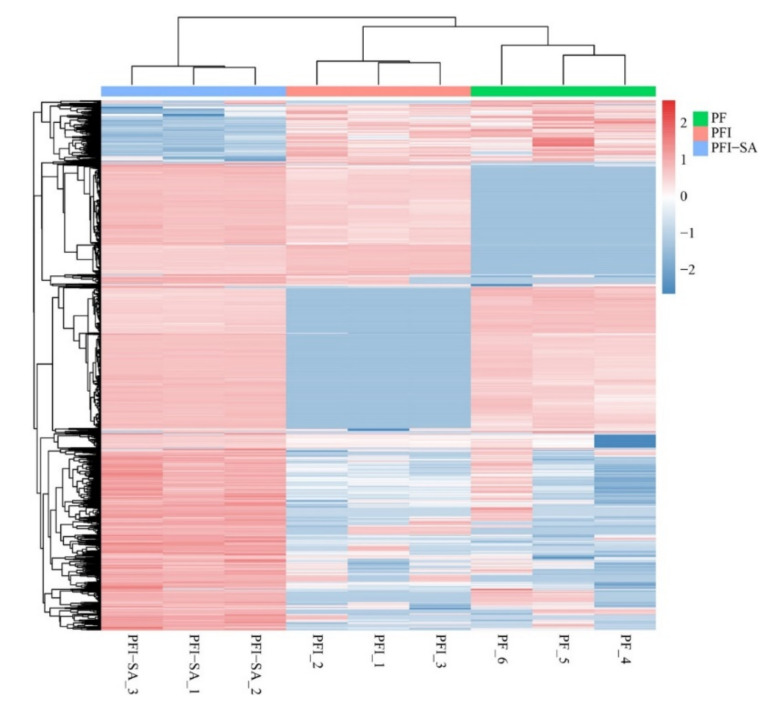
Cluster analysis of differentially expressed proteins (DEPs). PFI: *Paulownia fortunei* infected; PF: *Paulownia fortunei*.

In the PF vs. PFI group, using the MSstats 4.2.0 software package to analyze the proteomics data, differentially expressed proteins (DEPs) related to PaWB under SA treatment were identified (Figure 11). The DEPs were annotated to 46, 50, and 52 GO subcategories, with the highest number of proteins in catalytic activity in all three groups (Supplementary Figure S11). The classification of DEPs and the categories included were basically consistent. However, other organisms, parts of other organisms, and immune system processes were added in the groups after SA treatment. In all categories, the number of proteins in the SA treatment groups were generally higher than that in the PF vs. PFI group, indicating that the overall protein expression levels after SA treatment were higher. The most numerous categories in these two groups were catalytic activity and metabolic processes, suggesting that these two functions may play a key role in this biological process. The number of proteins in catalytic activity and metabolic processes after SA treatment was much higher than that in the untreated groups, indicating stronger metabolic and catalytic activity. Proteins involved in transcriptional regulatory activity, signal transduction, and antioxidant activity were also found to be significantly higher in the SA-treated groups (*p* ≤ 0.05). Moreover, there were also significant differences in the number of proteins related to cellular components such as cells, organelles, membranes, and extracellular regions, indicating a more rich and complete cellular structure in the cells after SA treatment.

These proteins mainly participate in important biological processes. The number of related proteins in the untreated groups was generally lower than that in the SA-treated groups, such as transport and metabolism classes, with only 17 proteins in the untreated group compared to 63 proteins in the SA-treated groups. This indicated that the biological processes in the SA-treated group were generally more active, and the differences in the metabolic category were the most significant. In addition, the number of proteins involved in lipid metabolism, amino acid metabolism, and carbohydrate metabolism in the untreated groups was much lower than that in the SA-treated groups, indicating that the material metabolism in the SA-treated groups was more active. In terms of gene information, the number of proteins related to translation, transcription, folding, sorting, and degradation in the untreated groups was also significantly lower than that in the SA-treated groups (*p* ≤ 0.05), indicating that protein processing and gene expression were more active after SA treatment (Supplementary Figures S12 and S13). Further screening for proteins related to the occurrence of PaWB identified two key significant DEPs, namely LTP family homologous to Arabidopsis LTP2 (Pfoxxg013220) and auxin protein (Pfo04g000940).

**Figure 12:**
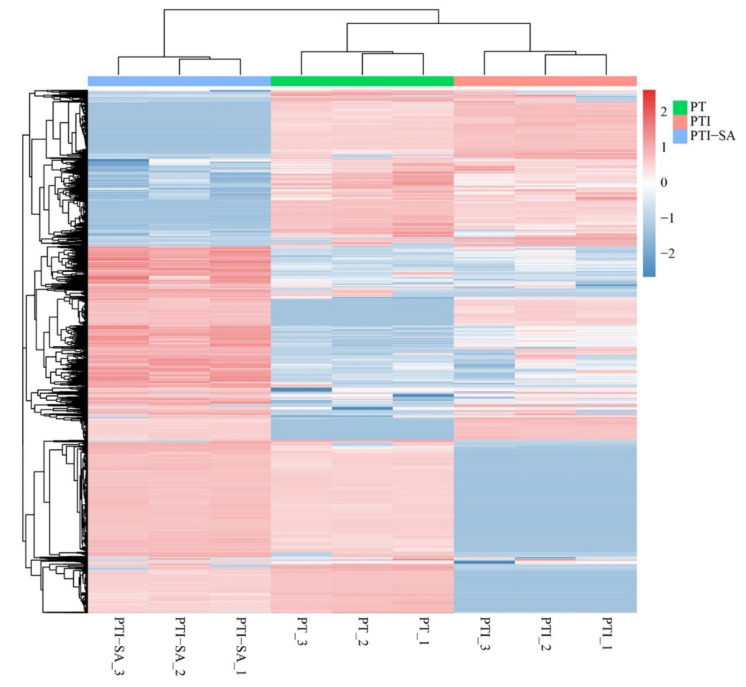
Cluster analysis of DEPs. PTI: *Paulownia tomentosa* infected; PT: *Paulownia tomentosa*.

In the PT vs. PTI group, the expression levels of DEPs were clustered (Figure 12). The functional characteristics of these DEPs were analyzed. The DEPs were annotated to 46, 51, and 53 GO subcategories, with the highest number of proteins in catalytic activity in all categories. In biological processes, most DEPs were enriched in cellular processes, metabolic processes, and biological regulation subcategories, indicating that SA-induced DEPs may be involved in regulating important cellular metabolism and physiological activities in Paulownia. In PT vs. PTI + SA, the result was significantly enriched in the biosynthesis of secondary metabolites and metabolic pathway of secondary metabolites, and it was also significantly enriched in the carbon metabolism pathways (Supplementary Figures S14–S16). The results showed a similar metabolic pathway enrichment pattern in PT. Metabolic pathways (2226, accounting for 27.58%), biosynthesis of secondary metabolites (1285, accounting for 15.92%), carbon metabolism (331, accounting for 4.1%), and protein processing in the endoplasmic reticulum (209, accounting for 2.59%) were enriched with a large number of DEPs, indicating that these biological processes may play a key role in the response of PT to SA. In the PTI vs. PTI + SA and PT vs. PTI groups, 176 significantly DEPs were screened, with 62 upregulated and 114 downregulated. In the PTI vs. PTI + SA and PT vs. PTI + SA groups, 373 significantly DEPs were screened, with 145 upregulated and 228 downregulated. Based on the analysis of the above two groups, four key DEPs were screened, including F-box protein (Pfo05g005630), CTP synthase N-terminus (Pfo09g000020), hydrolase folding protein homologous to Arabidopsis CXE1 (Pfo04g002250), and signal peptide peptidase gene SPPL2 protein (Pfo07g011870).

### 2.6. Transcriptomic and Proteomic Correlation Analysis

Through the analysis of DEGs and proteins at the transcriptomic and proteomic levels, it was found that metabolic pathways and the biosynthesis of secondary metabolites were the two most significant biological processes affected by SA-induced resistance against PaWB in PF and PT. These were mainly enriched in pathways such as the MAPK signaling pathway, biosynthesis of secondary metabolites, plant hormone signal transduction, and metabolic pathways. Further functional annotation of DEPs revealed a predominance of transcription factor families such as WRKY, CH3, MYB, NAC, and bHLH. This suggested that SA may influence the metabolic balance by regulating key enzymes in primary and secondary metabolism, thereby affecting its role in disease prevention and control.

Further screening of DEGs and proteins after SA treatment (log2 ≥ 3, *p* ≤ 0.05) revealed that three proteins were significantly upregulated, namely GHMP kinase C-terminus (Pfo16g005950), starch synthase catalytic domain (Pfo01g001190), and SacI homology domain (Pfo11g004680). The majority of the others were from the cytochrome P450 family, WRKY family, F-box family, and other key enzymes and regulatory factors.

## 3. Discussion

SA, as an endogenous plant signaling molecule, has been proven to induce systemic acquired resistance in plants. Studies have found that 2 mmol/L SA and 0.1 mmol/L methyl jasmonate significantly reduced the disease index of banana anthracnose [27]. Concentrations of 0.1 mmol/L and 0.2 mmol/L SA significantly enhanced wheat resistance to head blight and root rot [28]. Comparing the effects of SA on PF and PT seedlings with PaWB, it was found that the concentration of SA significantly affects the symptoms and growth of the plants. Despite PF and PT being different varieties, their response patterns to different concentrations of SA were very similar, suggesting that the optimal concentration of SA for alleviating PaWB varies little between different varieties, possibly due to a relatively conservative regulatory mechanism.

SA induces the expression of disease resistance-related proteins, accumulating pathogenesis-related proteins and enhancing POD and SOD activities and related gene expression in plants, thus strengthening the plant’s induced resistance [29]. This study found that SA treatment significantly increased the activities of POD and SOD in both types of Paulownia seedlings with PaWB, while significantly reduced the MDA content. This study also found that SA significantly promoted the deposition of callose in PFI and PTI. An analysis of callose suggested that SA may induce the synthesis and deposition of callose in PTI and PFI to enhance resistance to PaWB.

Transcriptomic and proteomic analyses screened out the MYB gene, Aux/IAA, BTB/POZ family, and cytochrome P450 family of genes related to hormone regulation and auxin signaling. Studies have shown that Arabidopsis AtMYB59 was induced by SA [30,31]. Moreover, PfBTB3/12/14/16/19/36/44 of the PfBTB family is associated with the occurrence of PaWB [32]. The expression levels of PfAux/IAA 13, PfAux/IAA 33, and PfAux/IAA 45 in seedlings with PaWB decreased after SA treatment. Moreover, SA response elements appear in the promoter regions of these genes, indicating their involvement in SA-induced defense responses [33]. After SA treatment, the expression of BRI1 in diseased and healthy Paulownia seedlings was upregulated, consistent with the trends in POD and SOD enzyme activities. DEGs such as RAX2 may play a key role in defense gene activation and SA signal transduction. After SA treatment, the expression of multiple proteins related to disease defense, signal transduction, protein synthesis, and modification in Paulownia was significantly upregulated. These DEPs may directly participate in the SA-induced disease resistance response in Paulownia. Comparing the DEPs in diseased and healthy seedlings before and after SA treatment showed significant differences, suggesting that the autonomous defense against PaWB in Paulownia and the SA-mediated disease resistance pathway may differ.

## 4. Materials and Methods

### 4.1. Experimental Materials

PF and PT, as well as PFI and PTI, were all provided by the Forest Biotechnology Laboratory of the Paulownia Research Institute at Henan Agricultural University in this study. These Paulownia seedlings were obtained through tissue culture techniques, with the following cultivation conditions: (25 ± 2) °C, light intensity of 130 μmol·m^−2^·s^−1^, and a photoperiod of 16 h [34]. The culture of the materials was conducted according to the method of Reference [35]. Apical buds from uniformly growing diseased seedlings were inoculated on 1/2 MS medium containing SA as the treatment group. Apical buds from healthy and diseased seedlings of both varieties were inoculated on 1/2 MS medium (without other hormones) as the control group. Each bottle contained 3 explants, with 30 bottles per treatment group, and the experiment was repeated 3 times.

### 4.2. Transcriptome Sequencing

Total RNA was extracted from SA-treated and untreated PF and PT infected with PaWB, as well as from healthy seedlings of both species. Each sample group had three biological replicates. Libraries were constructed using an mRNA enrichment method, followed by quantitative analysis to assess library quality [36]. DEGs were aligned with genes annotated in the GO and KEGG databases for sequence comparison and functional categorization. Using the R language platform DESeq2 (v1.4.5) and the Qvalue package RESM (v1.2.8), statistical tests were conducted on the enrichment of DEGs (*p*-value ≤ 0.05 and |log2FC| ≥ 1).

The original *p*-values were corrected for the False Discovery Rate (FDR), resulting in corrected *p*-values. After correction, the GO terms with *p*-value ≤ 0.05 were considered to be significantly enriched in functional items. Similarly, in KEGG enrichment analysis, metabolic pathways with a corrected *p*-value ≤ 0.05 were considered significantly enriched biological pathways. These enriched KEGG pathways reveal the key metabolic processes and signaling pathways regulated by DEGs. In this study, randomly selected DEGs were validated using qRT-PCR (Table 3).

### 4.3. Protein Identification

Peptides separated by liquid chromatography were ionized using a CSI nano source and then entered into a TimsTOF Pro tandem mass spectrometer for pattern detection. The retention time was calibrated using iRT peptides for DIA data. The false positives were controlled at an FDR of 1% based on the target–decoy model suitable for SWATH-MS.

Mass spectrometry data for 18 samples were collected in DIA mode using the TimsTOF Pro instrument. A protein database was constructed, and peptides and proteins were identified and quantified using MSstats software. The identified proteins were compared with the GO, KEGG, and COG databases for annotation information. Systematic errors in the samples were corrected, and normalization was performed. Finally, different proteins were evaluated for significant differences using a linear mixed-effects model based on the set comparison groups. Proteins with a |Fold change > 2| and *p*-value < 0.05 were selected as significantly different proteins.

### 4.4. DAB and NBT Staining

Fresh leaves from the same parts of different plants in both the treatment and control groups were collected and immersed in DAB and NBT staining solutions. After the staining reaction, the staining solution was discarded until the green pigment in the leaves completely faded to transparency. After decolorization, the leaf material was transferred to glycerin for preservation and observed under a microscope.

### 4.5. Aniline Blue Staining for Callose Detection

Fresh leaf tissues from the same parts of different plants treated with 0.1 mmol/L SA for 30 days and from the control group were soaked in FAA fixative for 24 h, then immersed in 70% and 50% anhydrous ethanol for 3 h each, followed by a 12 h soak in distilled water. The leaves were rinsed several times with distilled water and soaked in a 10% NaOH solution until transparent. Subsequently, the leaves were fully immersed in a working solution for callose staining. Using tweezers and a glass rod, the leaves were transferred to a slide to prepare leaf specimens, and the area and degree of callose accumulation were observed under a fluorescence microscope.

### 4.6. Measurement of Defensive Enzyme Activity

Leaf samples from both the experimental and control groups were taken, and the activities of POD, SOD, MDA, H_2_O_2_, and O_2_^−^—were measured using a micro-method according to the Solabio company’s reagent kit.

### 4.7. Detection of Phytoplasma

Total DNA from the apical buds of Paulownia tissue-cultured seedlings treated with SA and from the control group was extracted according to the following steps. Paulownia leaves were placed in a 2.0 mL centrifuge tube containing steel balls with a diameter of 5 mm, quickly frozen in liquid nitrogen for 5 min, and placed in a grinding machine for grinding samples until the leaves were ground to powder. Then, 800 µL SLS extract (Table 4 for preparation method) was added to the centrifuge tube, shaken for 5 min, and mixed upside down (vigorous action). Then, 800 µL (equal volume) 25:24:1 (Tris-equilibrium phenol: chloroform: isoamyl alcohol) was added, mixed, and shaken for 5 min. Then, the sample was centrifuged at 12,000 rpm/min at room temperature for 10 min, 500 µL of supernatant was absorbed into 1.5 mL centrifuge tube, isopropyl alcohol pre-cooled at −20 °C was added in equal volume, and the sample was mixed well. Then, the sample was centrifuged at 12,000 rpm/min at room temperature for 10 min and the supernatant was poured out; the bottom of the centrifuge tube had a white gel precipitate, this was rinsed with 500 µL 75% ethanol, centrifuged for 3 min, and the operation was repeated. Then, the supernatant was poured out, 500 µL of 100% anhydrous ethanol was added, the sample was left at room temperature for 5 min, centrifuged for 2 min, the supernatant was poured out, and the sample was left to dry. After adding 300 µL ddH_2_O dissolved DNA, it can be stored at −20 °C for a long time. Finally, the concentration and purity of the total DNA samples were detected by NanoDrop2000 and 1% agar gel electrophoresis. Phytoplasma was amplified by two rounds of PCR using the phytoplasma 16S rDNA primers R16mF1/R16mR1 and R16F2/R16R2. Nested PCR was then performed using this DNA as a template to detect the presence of phytoplasma in the Paulownia samples.

### 4.8. Data Processing and Statistical Analysis

All physiological, biochemical, and defense-related measurements in this experiment were derived from the average of three biological replicates. All data were organized using Excel and analyzed for significant differences using SPSS. Graphs were generated using GraphPad Prism 9.0 software.

## 5. Conclusions

In this work, the preventive and therapeutic effects of SA on PaWB and its effects on the transcriptome and proteome were studied. The results demonstrated that the optimal SA concentration for inducing resistance to PaWB was 0.10 mmol/L. After SA treatment, the phytoplasma content in diseased seedlings was reduced compared to the control group, indicating that SA treatment could reduce the amount of phytoplasma. Moreover, after exogenous SA treatment, the activities of POD and SOD in PFI and PTI were enhanced, and the contents of MDA, H_2_O_2_, and O_2_^−^ were significantly reduced. Callose accumulation occurred in the leaves with PaWB, and its content significantly increased after SA treatment. The DEGs were mainly enriched in the MAPK plant signaling pathway and plant hormone signal transduction pathway. Transcription factor genes such as WRKY and MYB, as well as genes involved in the SA-mediated defense response against PaWB, such as RAX2, showed significant differences. Functional classification analysis of the DEPs suggested that these proteins were mainly involved in processes such as disease defense, signal transduction, transcription, and protein synthesis in PF and PT. After SA treatment, proteins involved in signal transduction, protein synthesis modification, and disease defense were differentially expressed.

## Figures and Tables

**Table 1:** Transcriptome sequencing data statistics table.

Sample	Total Raw Reads (M)	Total Clean Reads (M)	Total Clean Bases (Gb)	Clean Reads Q20 (%)	Clean Reads Q30 (%)	Clean Reads Ratio (%)
PF_1	42.64	42.64	6.4	98.33	94.36	100
PF_2	42.51	42.51	6.38	98.41	94.41	100
PF_3	42.53	42.53	6.38	98.58	94.66	100
PFI + SA_1	43.69	42.09	6.31	98.17	94.08	96.34
PFI + SA_2	43.69	42.27	6.34	98.37	94.66	96.74
PFI + SA_3	43.69	42.28	6.34	98.37	94.65	96.76
FI_1	42.51	42.51	6.38	98.74	95.31	100
PFI_2	42.78	42.78	6.42	98.65	94.94	100
PFI_3	42.41	42.41	6.36	98.59	94.73	100
PT_1	42.62	42.62	6.39	98.43	94.1	100
PT_2	42.63	42.63	6.4	98.46	94.25	100
PT_3	42.66	42.66	6.4	98.55	94.55	100
PTI + SA_1	43.69	42.43	6.36	98.47	94.97	97.11
PTI + SA_2	43.69	42.38	6.36	98.36	94.6	97
PTI + SA_3	43.69	42.22	6.33	98.47	94.97	96.63
PTI_1	42.68	42.68	6.4	98.44	94.14	100
PTI_2	42.35	42.35	6.35	98.43	94.09	100
PTI_3	42.3	42.3	6.34	98.42	94.06	100

**Table 2:** Proteomic analysis under SA treatment of the two screened *Paulownia* species.

Analysis Group	Downregulated	Upregulated
PFI vs. PF	267	199
PFI + SA vs. PF	519	1156
PFI + SA vs. PFI	403	1122
PTI vs. PT	236	544
PTI + SA vs. PT	744	1602
PTI + SA vs. PTI	589	1035

**Table 3:** qRT-PCR primers in this study.

Gene ID	Forward Primer (5′–3′)	Reverse Primer (5′–3′)
*Pfo09g004910*	AAGCGAAGGAAGAGTCAGGC	CGCCGAACAACCCTTTGAAC
*Pfo13g007180*	GAGCACTTATGCCCACGAGA	TCAGGGAAGCTGTTGTTGCT
*Pfo11g014070*	GACGACGACCCCACCATATT	TCCAAGTCCTCGGTGTTGAC
*Pfo04g009890*	TGCTGGCGGCATAACATTGA	GCCCACATCCAACGACAAGT
*Pfo06g001070*	ACCATTGACTGATGCAGCGA	GAGGCCACCTGAAGGTCATC
*Pfo14g010380*	GGCTGATGCAGTTCAACCAC	CCTAGTCCACTTCAGCGCAA
*Pfo15g012670*	CAGTTGCTTGCAGGCTTGAC	TCATAATCCGCTGCACGGTC
*Pfo19g006230*	TGTGCAGCTAGATGTGGCAA	GCAACTCTCTTCAAGCGTCC
*PfActin*	AATGGAATCTGCTGGAAT	ACTGAGGACAATGTTACC

**Table 4:** Preparation method of SLS extraction solution.

Regent	100 mL Use Quantity (g)	Method
0.2 M EDTA-Na_2_	7.4	Add water and set the volume to 100 mL, then add about 0.8 g NaOH, and adjust the pH to about 8.0
1 M Tris-Base	12.1	Add water and set the volume to 100 mL, then add about 4.2 mL concentrated HCl, and adjust the pH to about 8.0
1 M NaCl	5.85	Add water and set the volume to 100 mL

## Data Availability

The original contributions presented in the study are included in the article/Supplementary Materials, further inquiries can be directed to the corresponding author/s.

## Supplementary Materials

The following supporting information can be downloaded at https://www.mdpi.com/article/10.3390/ijms251910553/s1.
