# Supplementary material for: Novel Insight into the Prevention and Therapeutic Treatment of Paulownia Witches’ Broom: A Study on the Effect of Salicylic Acid on Disease Control and the Changes in the Paulownia Transcriptome and Proteome
Source: Int J Mol Sci. 2024 Sep 30;25(19):10553. doi: 10.3390/ijms251910553 (PMC11476412; doi:10.3390/ijms251910553)
Supplement: Supplementary file 1 [file ijms-25-10553-s001.zip › ijms-3143401-supplementary.pdf]

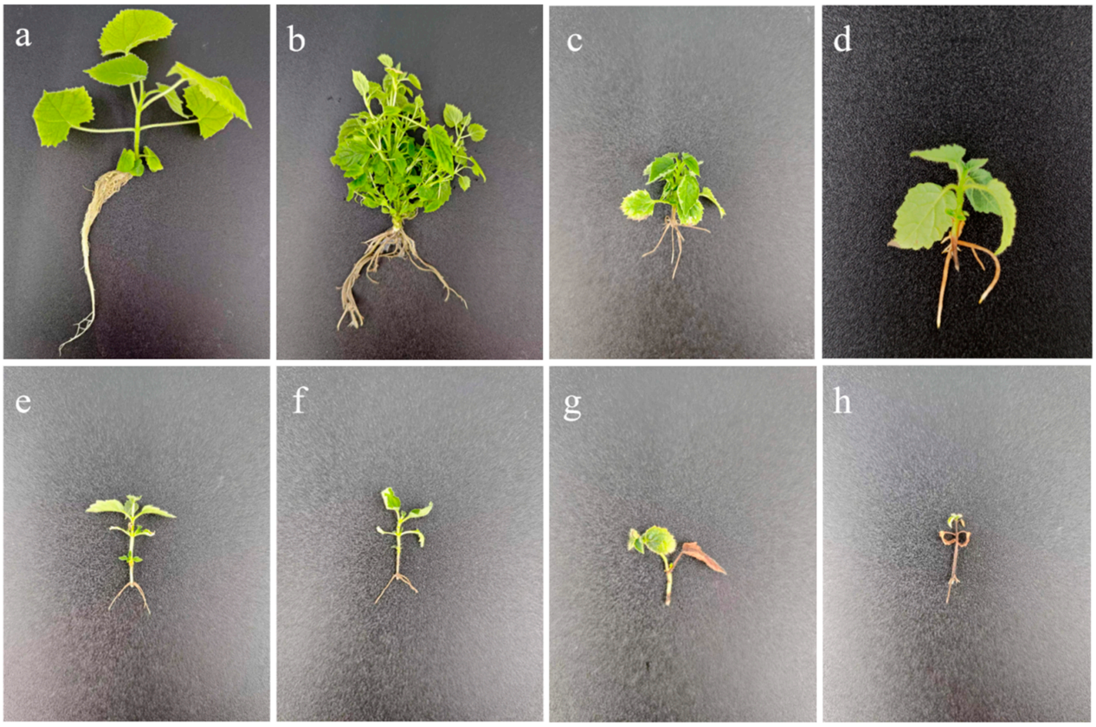

Supplementary Figure S1. SA treatment effects on the growth morphology of *Paulownia foutunei* (PF) seedlings and *Paulownia foutunei* infected (PFI) seedlings. **(a)** PF healthy seedlings. **(b)** PFI seedlings. **(c)** PFI+0.05 mM SA treatment. **(d)** PFI+0.10mM SA treatment. **(e)** PFI+0.15 mM SA treatment. **(f)** PFI+0.20 mM SA treatment. **(g)** PFI+0.25 mM SA treatment. **(h)** PFI+0.30 mM SA treatment.

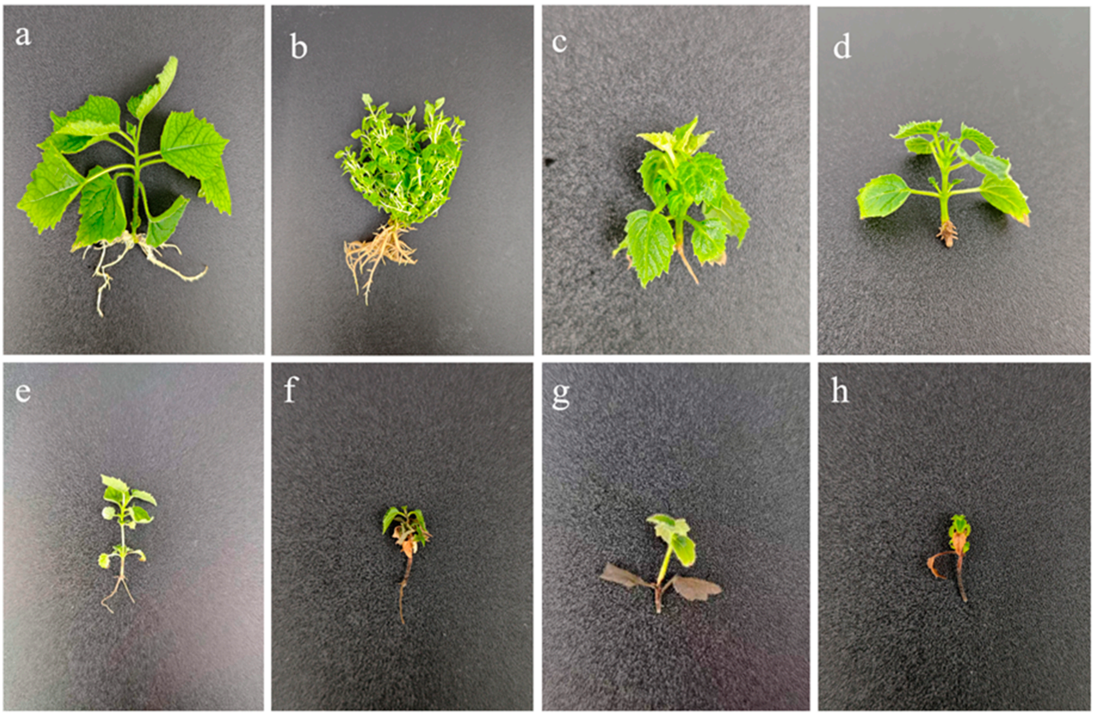

Supplementary Figure S2. SA treatment effects on the growth morphology of *Paulownia tomentosa* (PT) seedlings and *Paulownia tomentosa* infected (PTI) seedlings. **(a)** PT healthy seedlings. **(b)** PTI seedlings. **(c)** PTI+0.05 mM SA treatment. **(d)** PTI+0.10mM SA treatment. **(e)** PTI+0.15 mM SA treatment. **(f)** PTI+0.20 mM SA treatment. **(g)** PTI +0.25 mM SA treatment. **(h)** PTI +0.30 mM SA treatment.

A

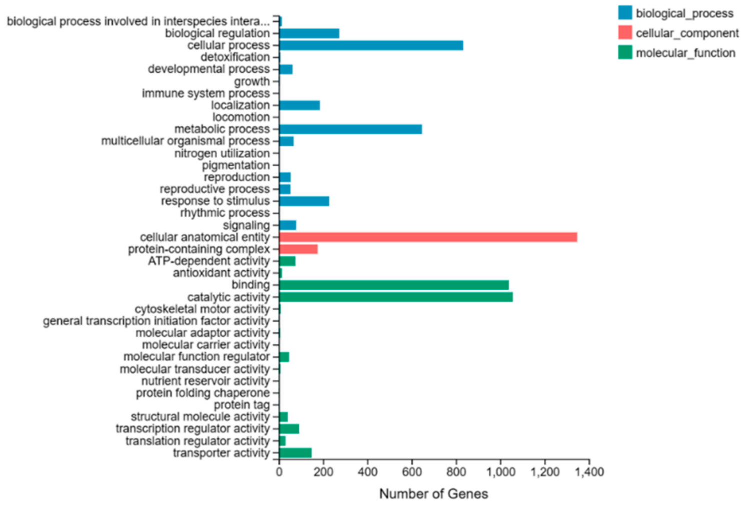

B

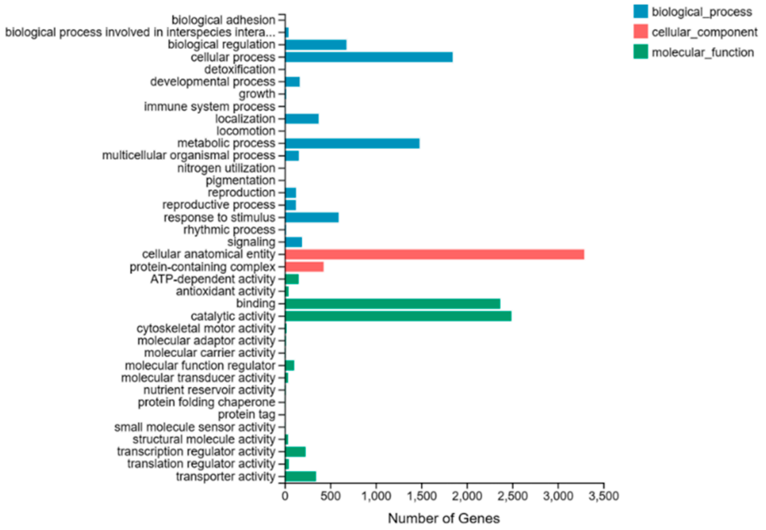

C

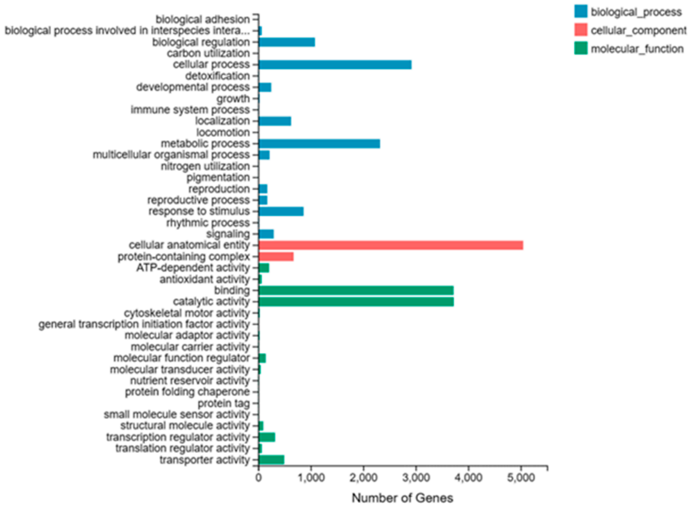

Supplementary Figure S3. GO classification of DEGs of *Paulownia foutunei* (PF) and *Paulownia foutunei* infected (PFI). (A) PF vs PFI. (B) PFI vs PFI+SA. (C) PF vs PFI+SA.

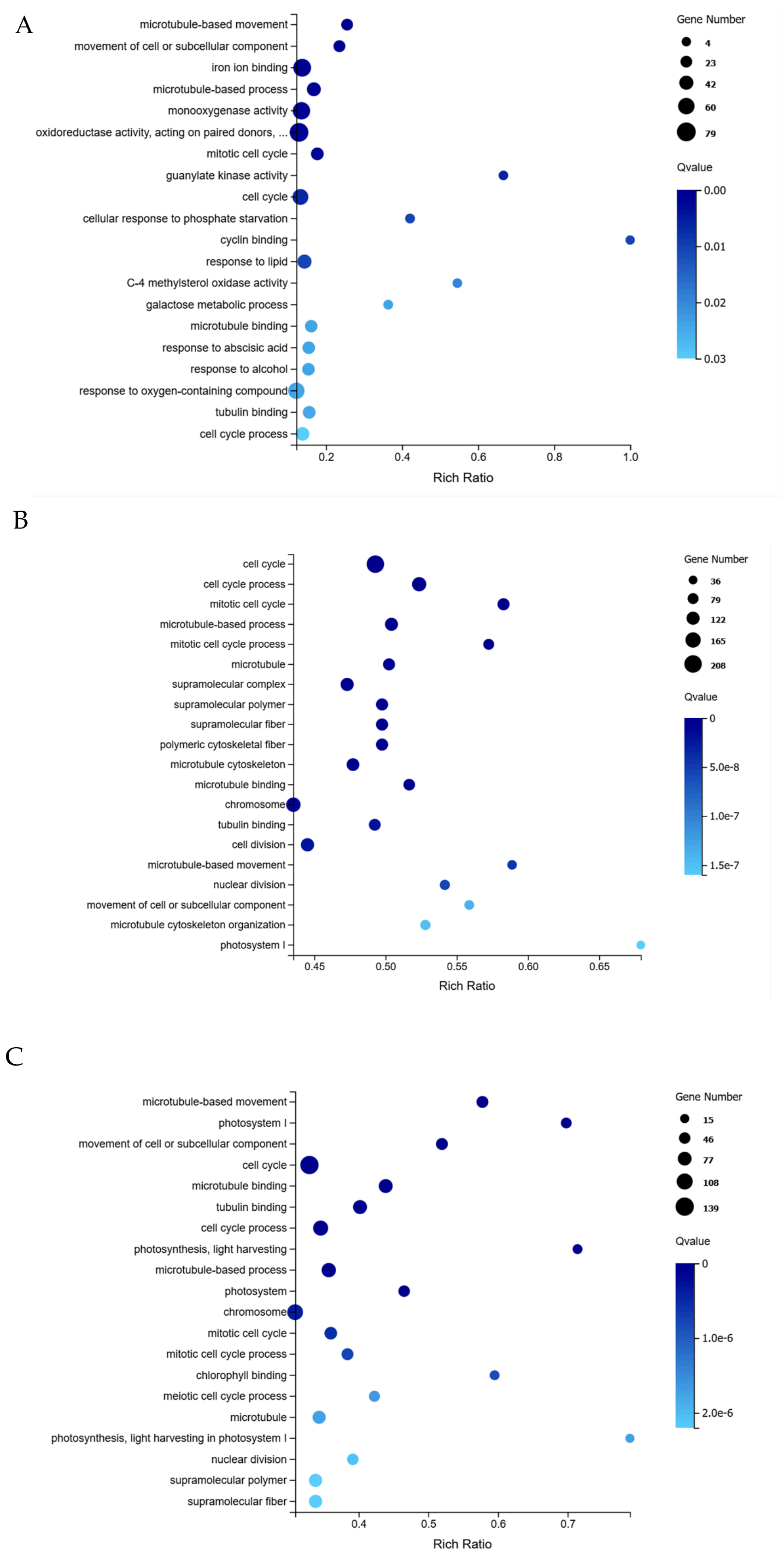

Supplementary Figure S4. GO beneficiation of DEGs of *Paulownia foutunei* (PF) and *Paulownia foutunei* infected (PFI). (A) PF vs PFI. (B) PFI vs PFI+SA. (C) PF vs PFI+SA.

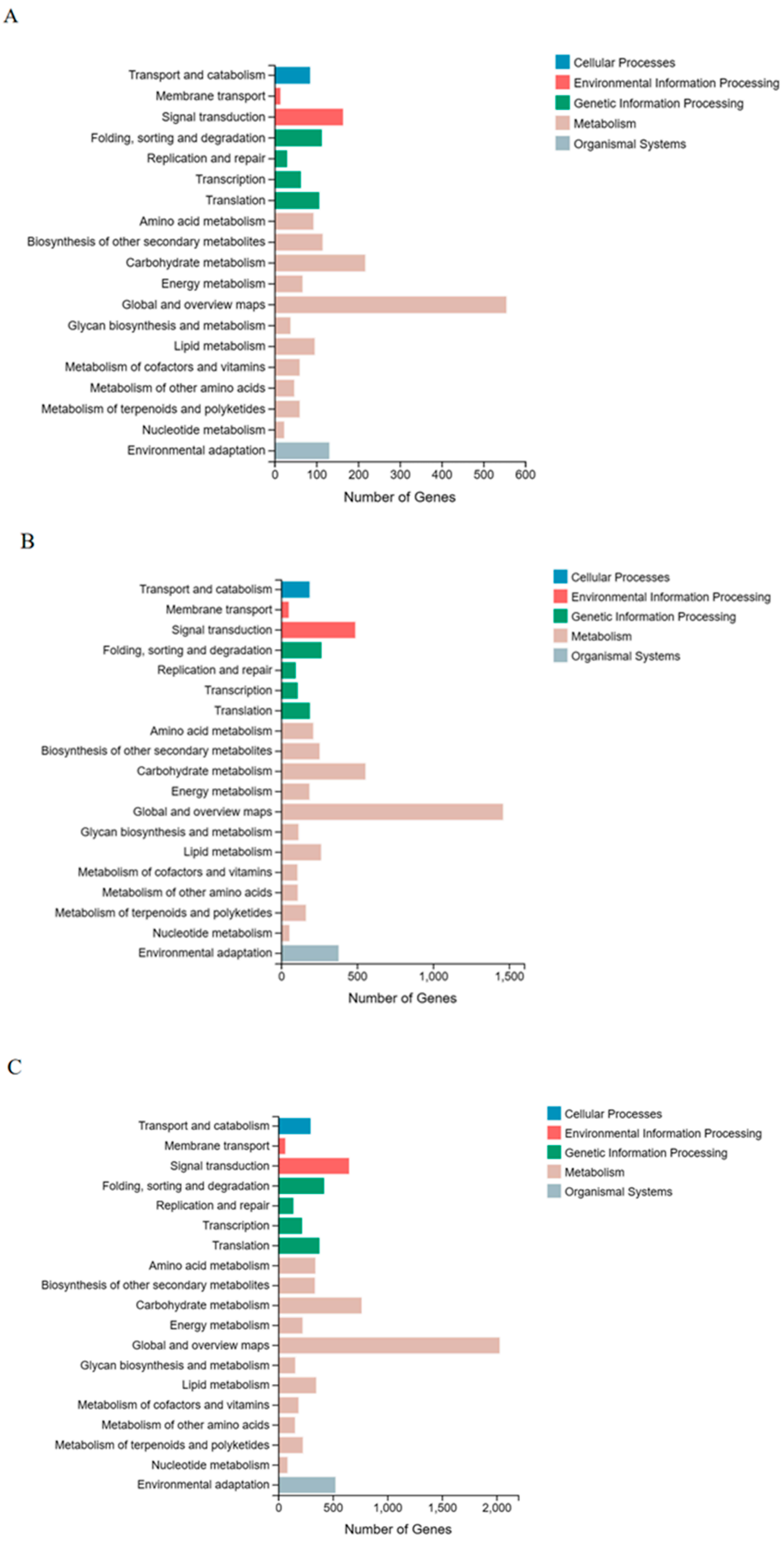

Supplementary Figure S5. KEGG enrichment classification of DEGs of *Paulownia foutunei* (PF) and *Paulownia foutunei* infected (PFI). (A) PF vs PFI. (B) PFI vs PFI+SA. (C) PF vs PFI+SA.

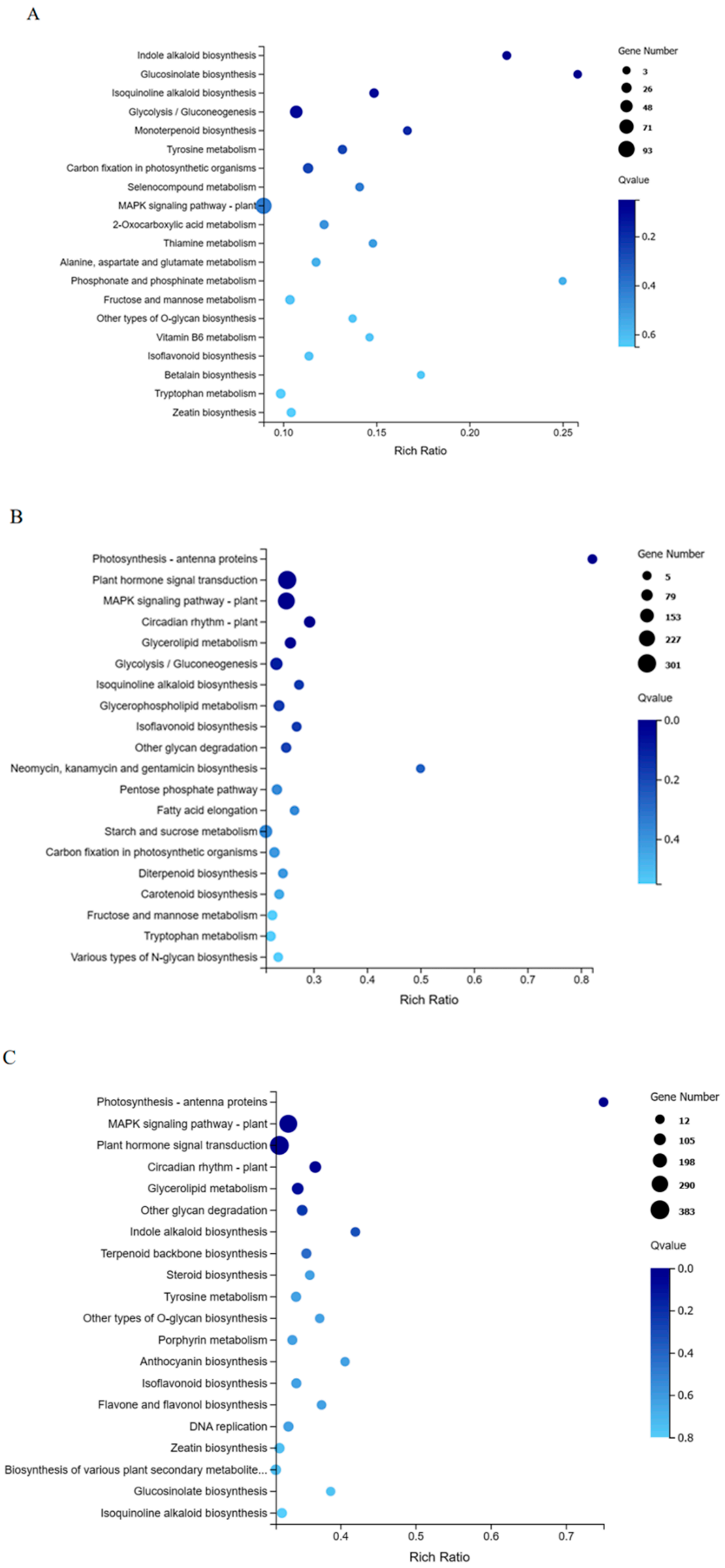

Supplementary Figure S6. Bubble map of KEGG enrichment of DEGs in *Paulownia foutunei* (PF) and *Paulownia foutunei* infected (PFI). (A) PF vs PFI. (B) PFI vs PFI+SA. (C) PF vs PFI+SA.

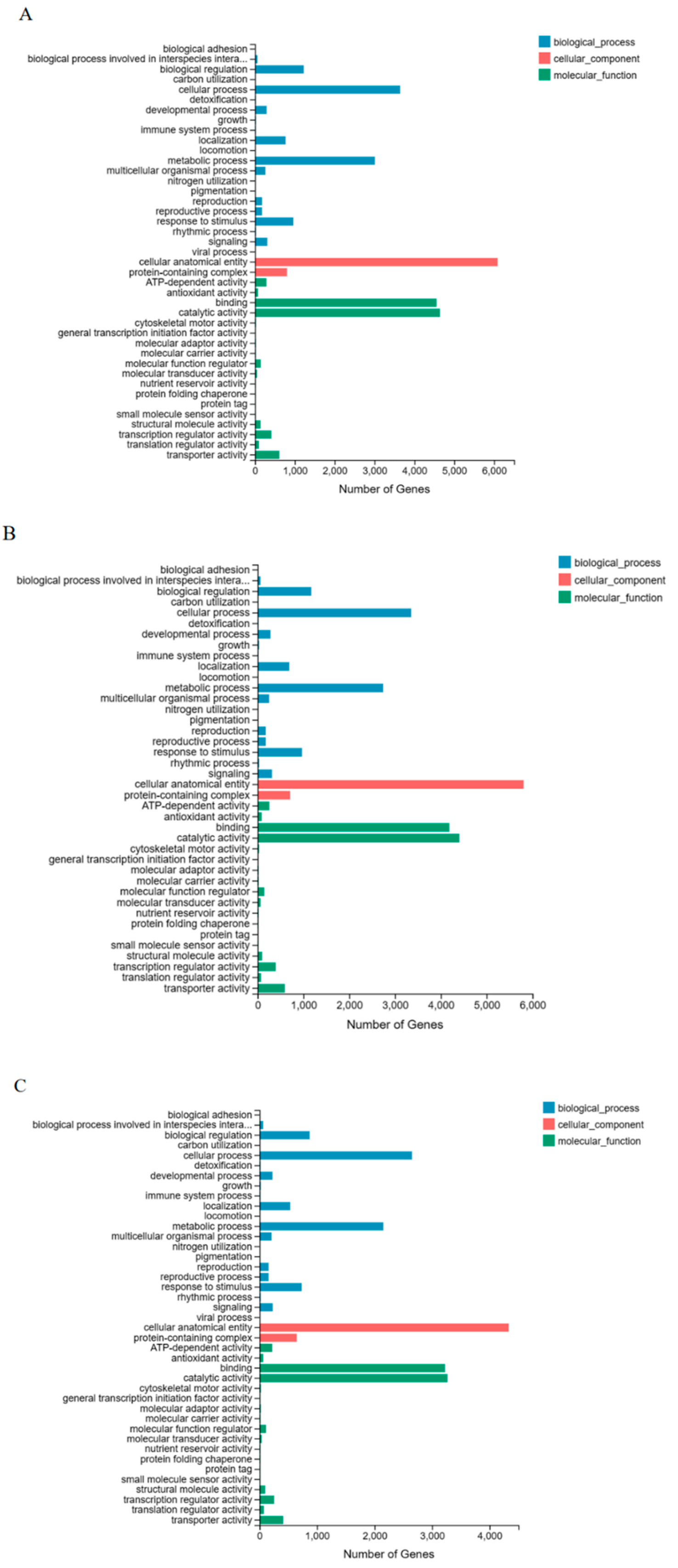

Supplementary Figure S7. GO classification of DEGs of *Paulownia tomentosa* (PT) and *Paulownia tomentosa* infected (PTI). (A) PT vs PTI. (B) PTI vs PTI+SA. (C) PT vs PTI+SA.

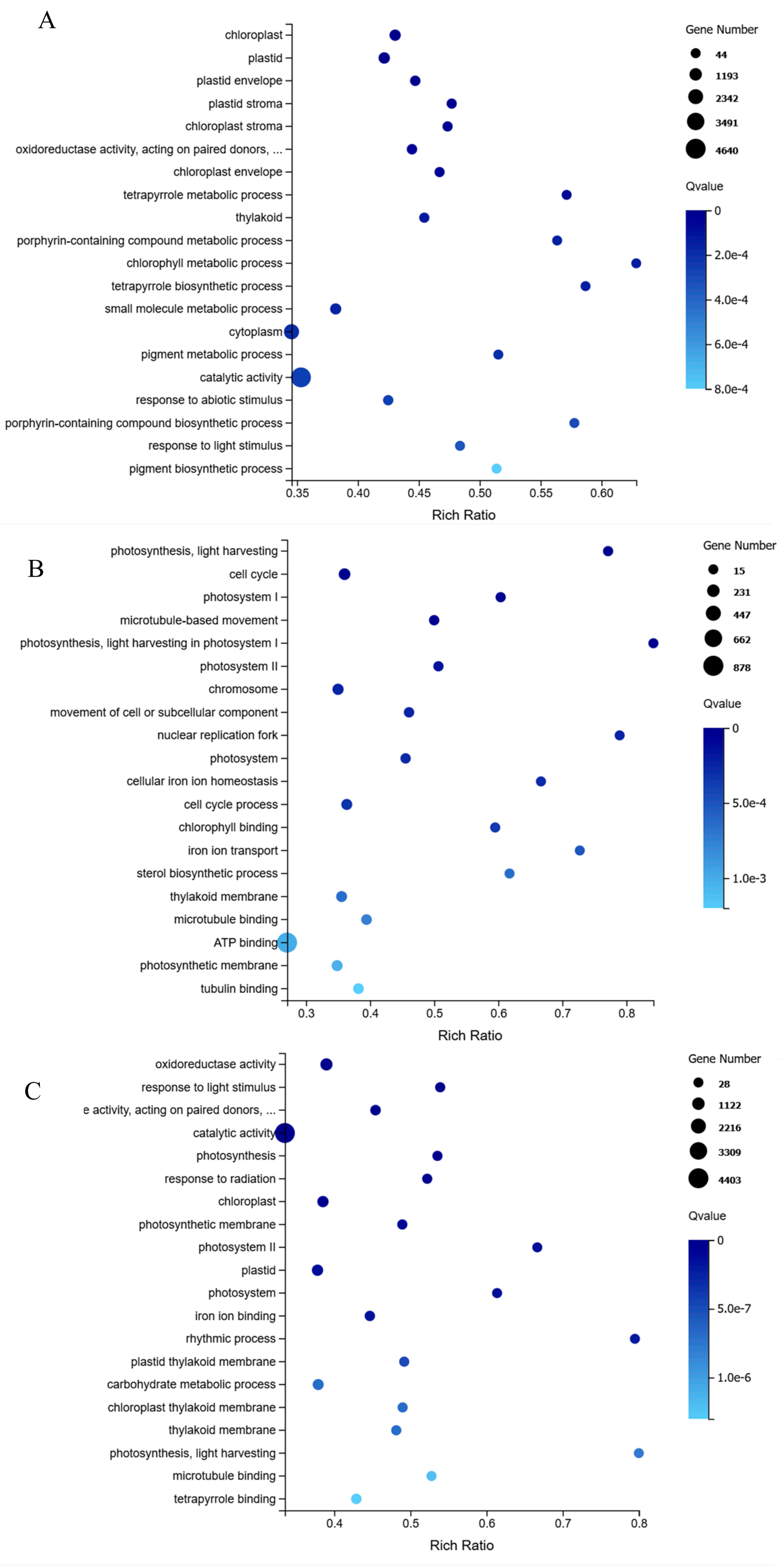

Supplementary Figure S8. GO beneficiation of DEGs of *Paulownia tomentosa* (PT) and *Paulownia tomentosa* infected (PTI). (A) PT vs PTI. (B) PTI vs PTI+SA. (C) PT vs PTI+SA.

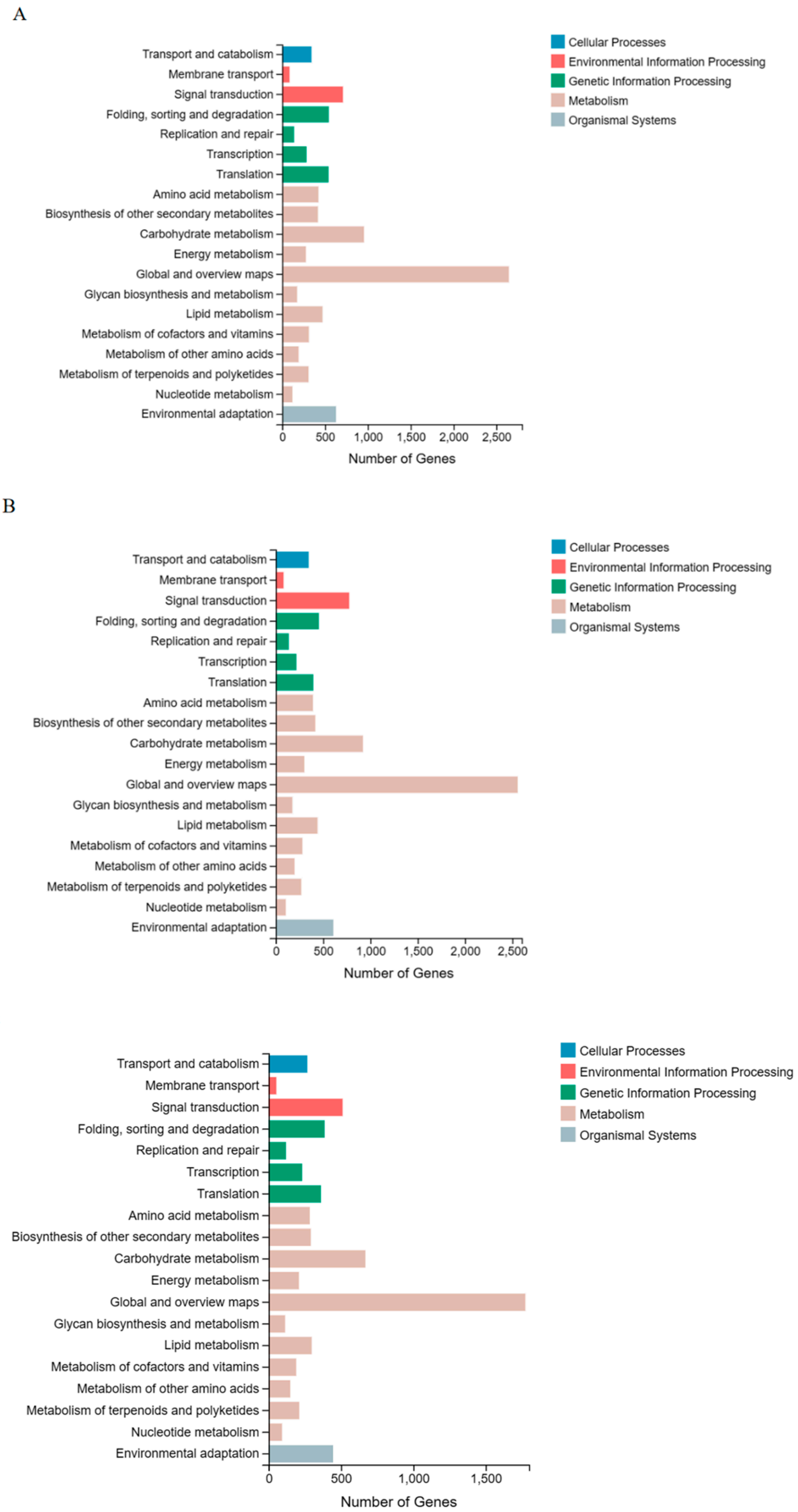

Supplementary Figure S9. KEGG enrichment classification of DEGs of *Paulownia tomentosa* (PT) and *Paulownia tomentosa* infected (PTI). (A) PT vs PTI. (B) PTI vs PTI+SA. (C) PT vs PTI+SA.

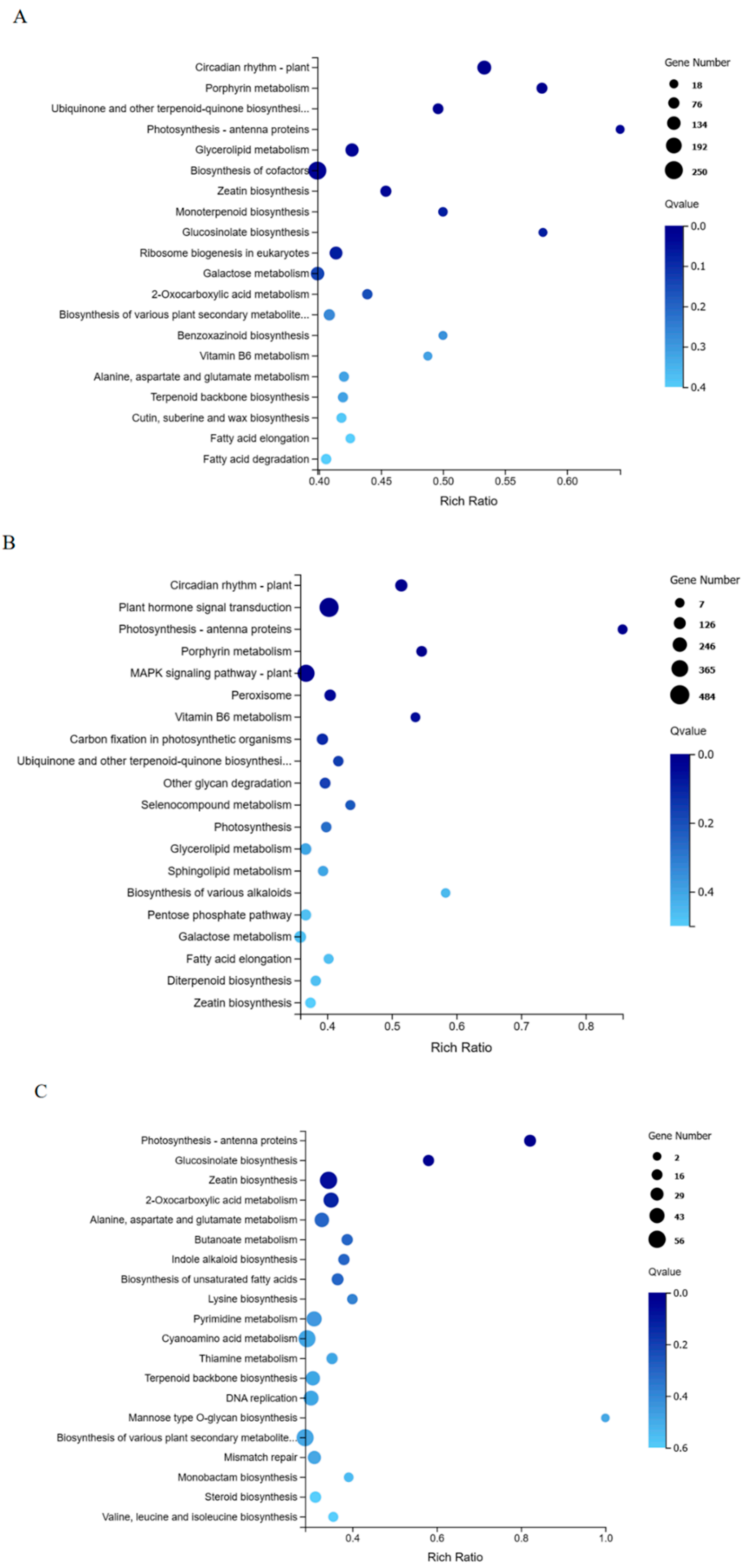

Supplementary Figure S10. Bubble map of KEGG enrichment of differential genes in *Paulownia tomentosa* (PT) and *Paulownia tomentosa* infected (PTI). (A) PT vs PTI. (B) PTI vs PTI+SA. (C) PT vs PTI+SA.

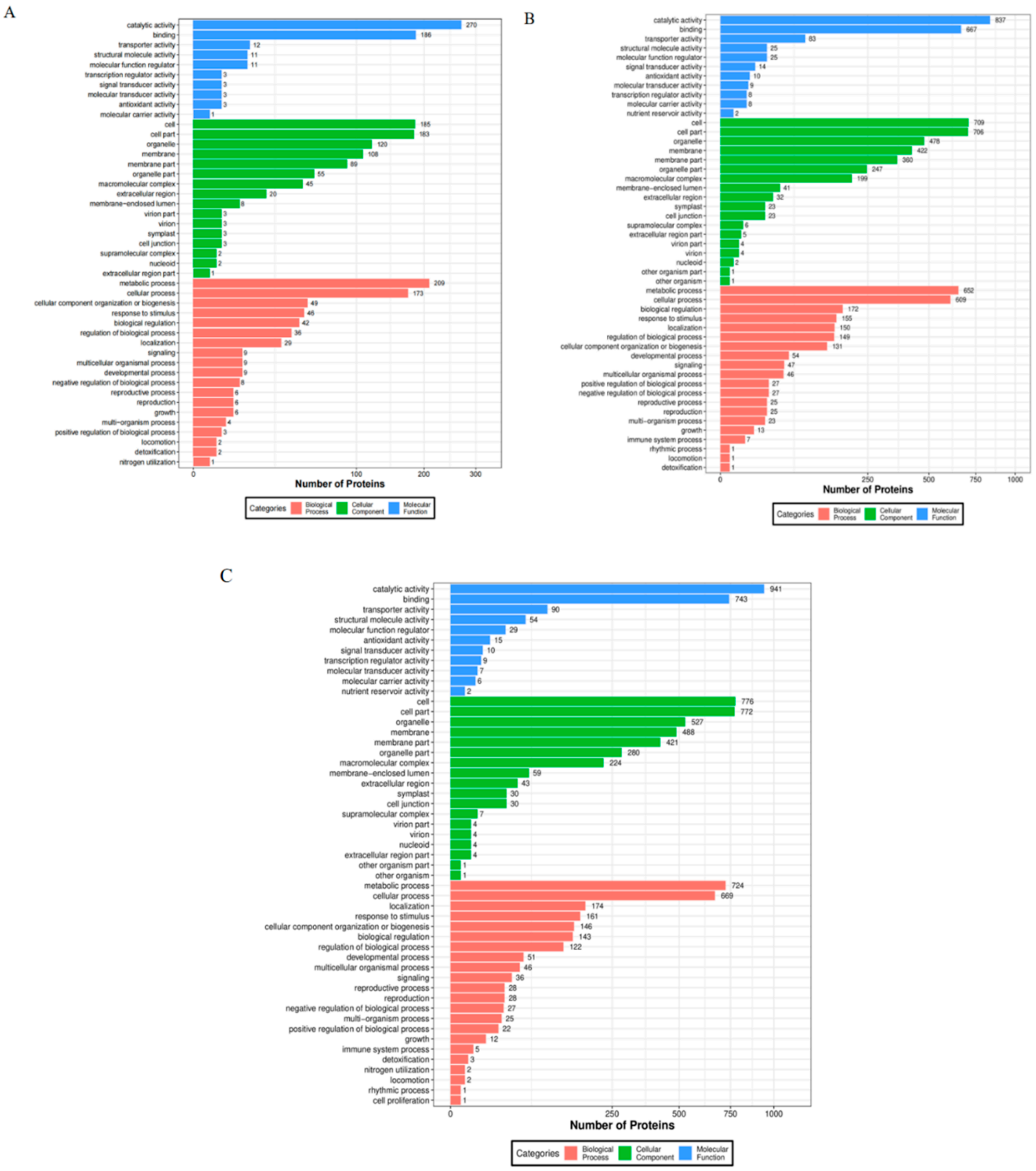

Supplementary Figure S11. GO classification of differentially expressed proteins (DEPs) of *Paulownia foutunei* (PF) and *Paulownia foutunei* infected (PFI). (A) PF vs PFI. (B) PFI vs PFI+SA. (C) PF vs PFI+SA.

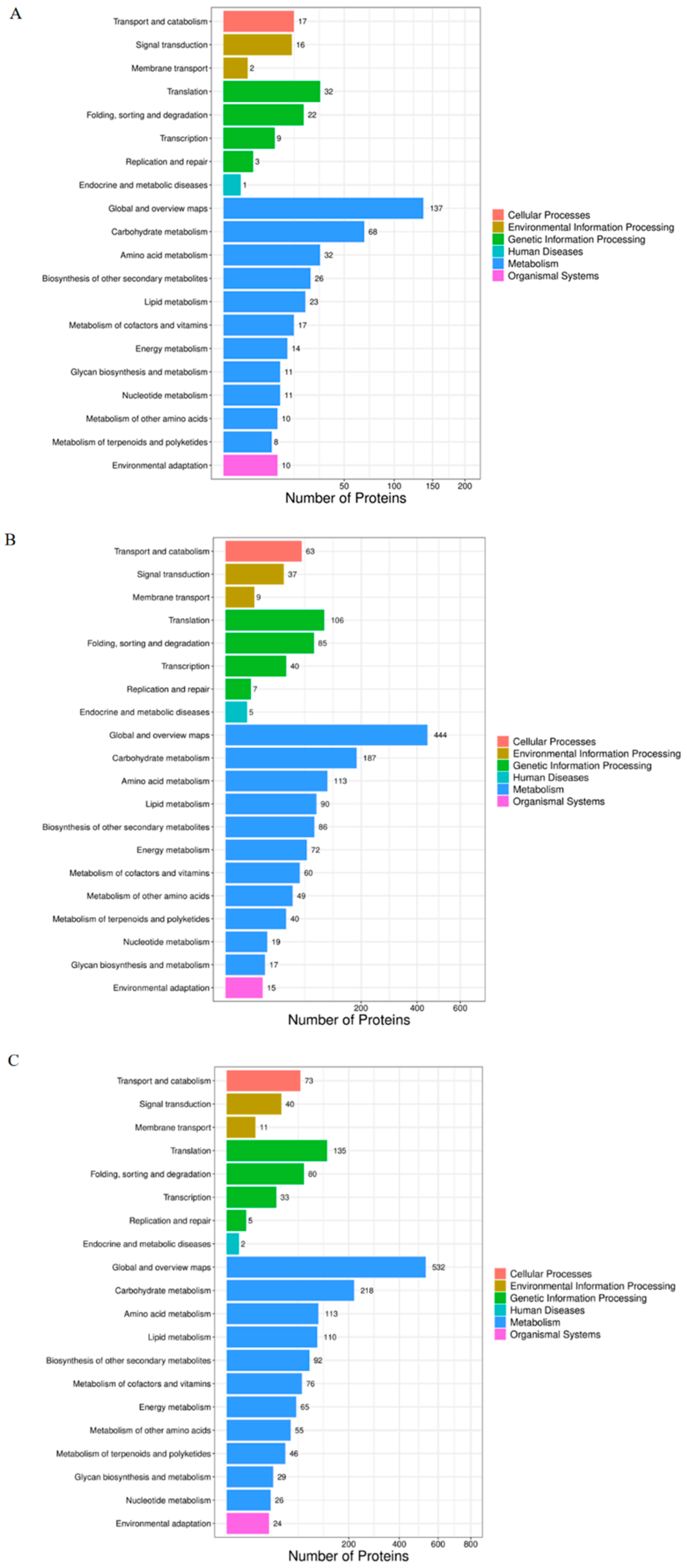

Supplementary Figure S12. KEGG enrichment classification of DEPs of *Paulownia foutunei* (PF) and *Paulownia foutunei* infected (PFI). (A) PF vs PFI. (B) PFI vs PFI+SA. (C) PF vs PFI+SA.

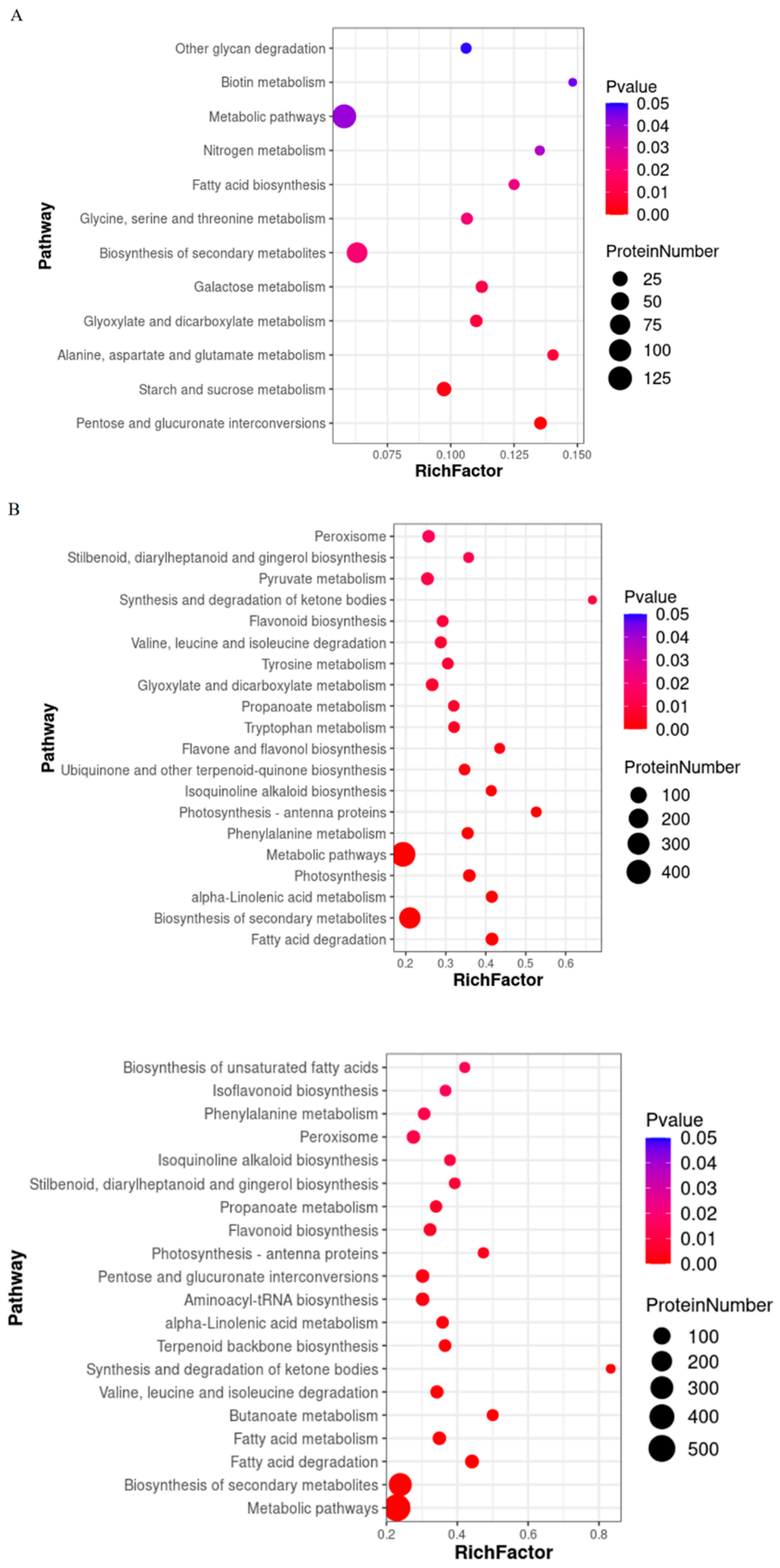

Supplementary Figure S13. Bubble map of KEGG enrichment of DEPs in *Paulownia foutunei* (PF) and *Paulownia foutunei* infected (PFI). (A) PF vs PFI. (B) PFI vs PFI+SA. (C) PF vs PFI+SA.

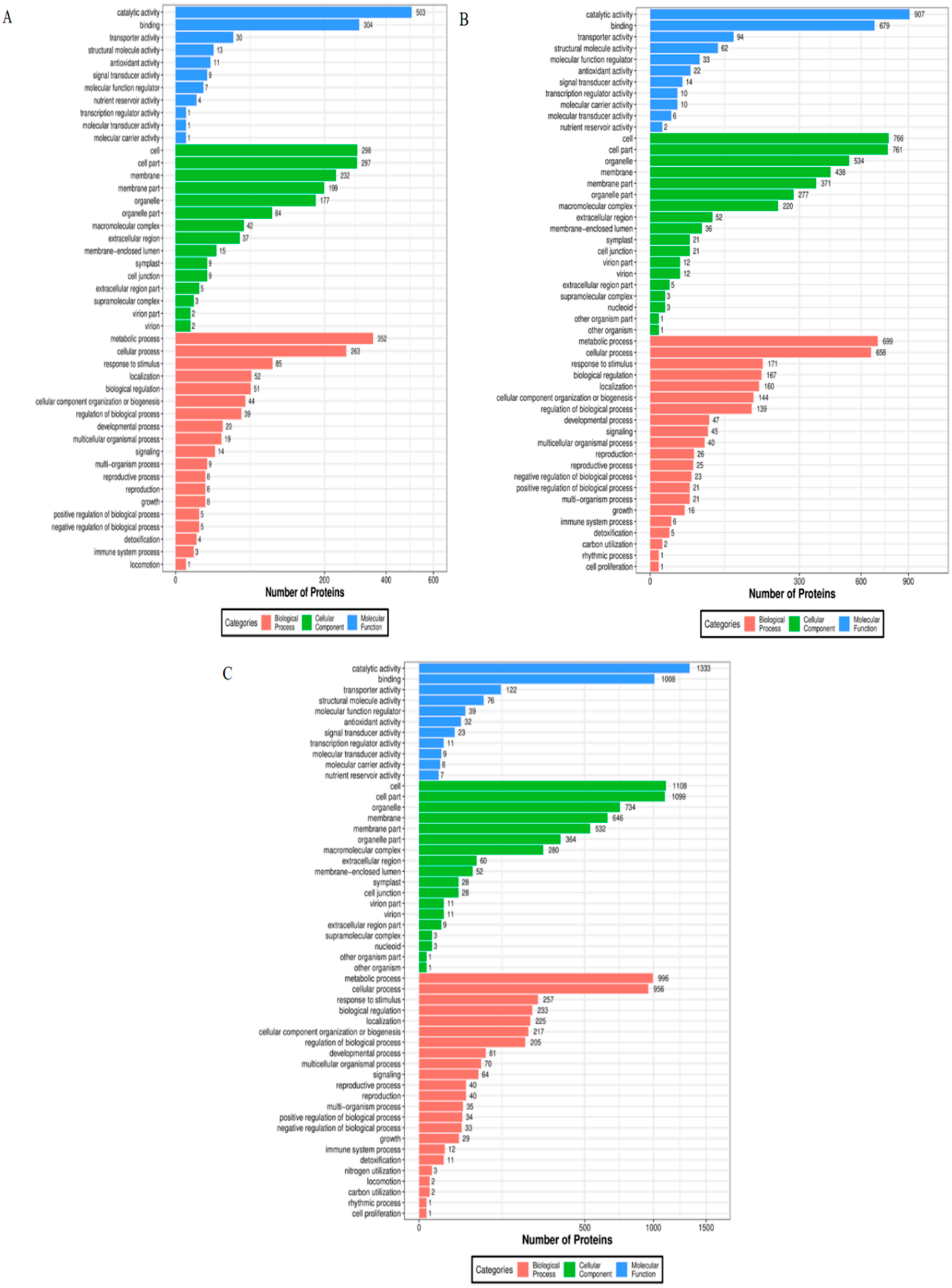

Supplementary Figure S14. GO classification of DEPs of *Paulownia tomentosa* (PT) and *Paulownia tomentosa* infected (PTI). (A) PT vs PTI. (B) PTI vs PTI+SA. (C) PT vs PTI+SA.

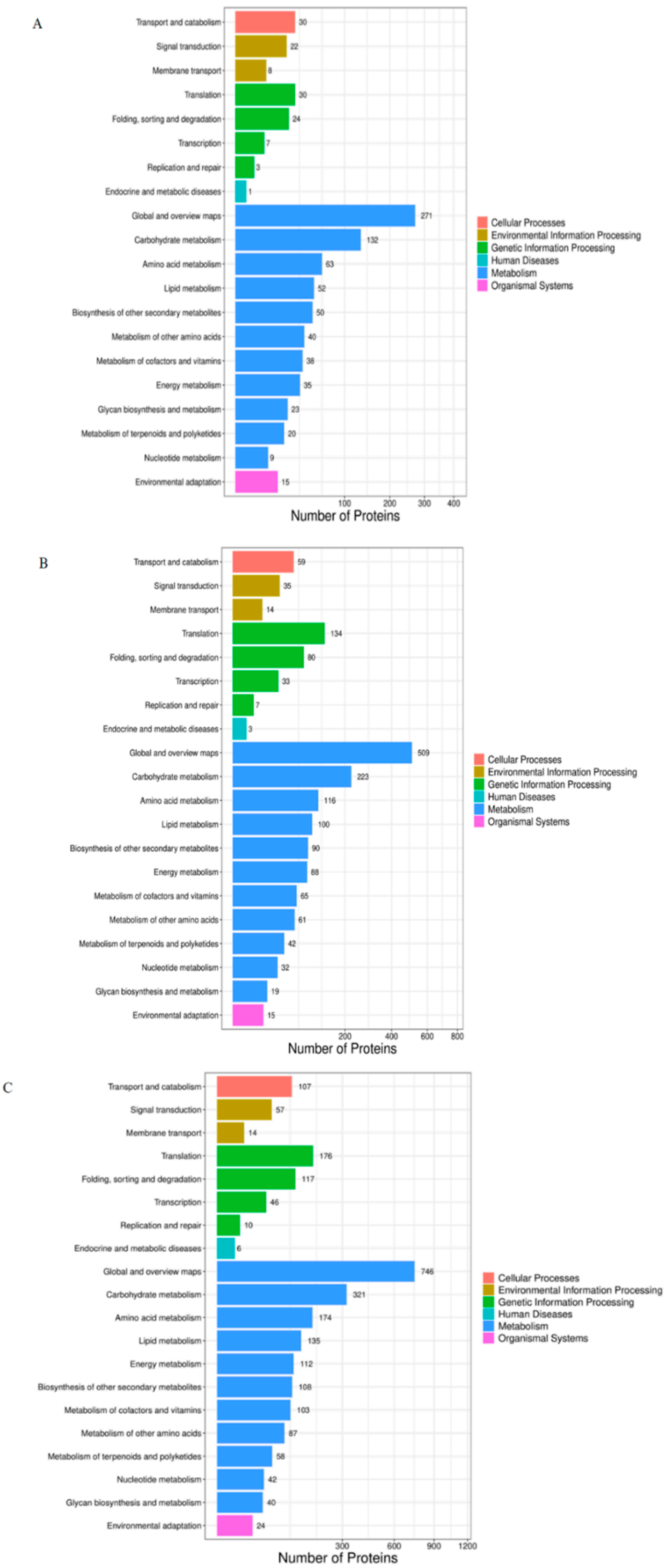

Supplementary Figure S15. KEGG enrichment classification of DEPs of *Paulownia tomentosa* (PT) and *Paulownia tomentosa* infected (PTI). (A) PT vs PTI. (B) PTI vs PTI+SA. (C) PT vs PTI+SA.

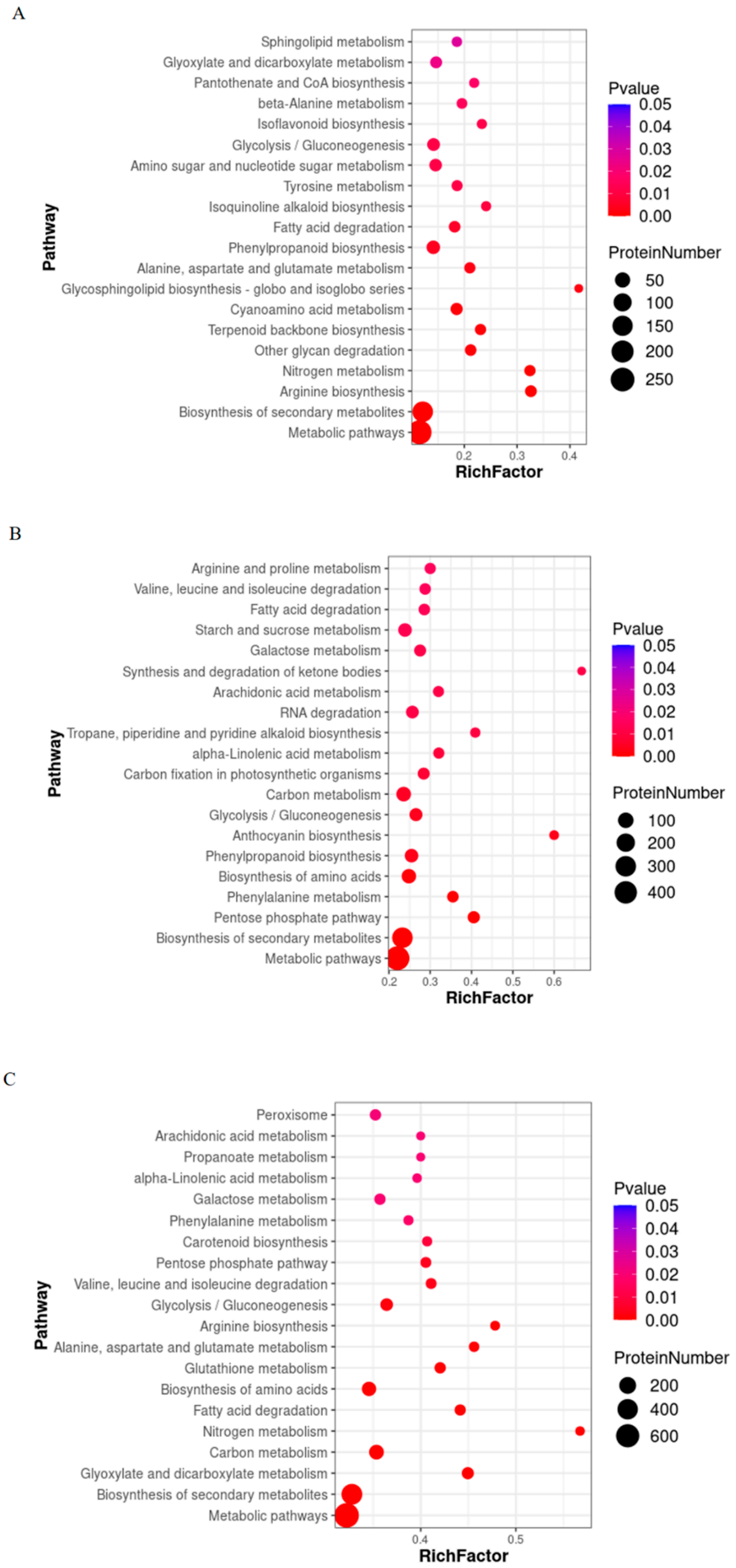

Supplementary Figure S16. Bubble map of KEGG enrichment of DEPs in *Paulownia tomentosa* (PT) and *Paulownia tomentosa* infected (PTI). (A) PT vs PTI. (B) PTI vs PTI+SA. (C) PT vs PTI+SA.
